# Supplementary material for: Noncanonical circRNA biogenesis driven by alpha and gamma herpesviruses
Source: EMBO J. 2025 Mar 3;44(8):2323–52. doi: 10.1038/s44318-025-00398-0 (PMC12000468; doi:10.1038/s44318-025-00398-0)
Supplement: Supplementary file 1 — Appendix [file 44318_2025_398_MOESM1_ESM.pdf]

Appendix for

Noncanonical circRNA biogenesis driven by alpha and gamma herpesviruses

Table of contents:

Appendix Table S1. RNA-Seq data summary for lytic, latent, and reactivation models	Page 2
Appendix Table S2. High confidence circRNAs in lytic, latent, and reactivation models	Page 2
Appendix Figure S1. Viral gene expression in HSV-1, KSHV, and MHV68 models	Page 3
Appendix Figure S2. Reproducibility of high confidence circRNA calls	Page 4
Appendix Figure S3. Divergent primer validation	Page 5-6
Appendix Figure S4. CircRNA cis-element analysis for additional lytic infection models	Page 7
Appendix Figure S5. Protein levels of predicted RBP-circRNA partners during infection	Page 8
Appendix Figure S6. Impact of spliceosome inhibition on infection models	Page 9
Appendix Figure S7. ddPCR quantitation of HSV-1 RNAs after spliceosome inhibition	Page 10-11
Appendix Figure S8. Impact of spliceosome depletion on KSHV circRNA levels	Page 12
Appendix Figure S9. RNA ligase expression following siRNA depletion	Page 13
Appendix Figure S10. circRNAs unaffected by RNA ligase depletion	Page 14
Appendix Figure S11. HSV-1 and KSHV gene expression in mutant viral models	Page 15
Appendix Figure S12. MHV68 gene expression in mutant viral models	Page 16
Appendix Figure S13. HSV-1 and KSHV transcript profiles in mutant viral models	Page 17
Appendix Figure S14. ORF57 eCLIP heatmap	Page 18
Appendix Figure S15. ORF57 regulation of linear viral transcripts	Page 19
Appendix Figure S16. ORF57 dependence of PAN BSJ variants	Page 20

Appendix Table S1. RNA-Seq data summary for lytic, latent, and reactivation models.

Total mapped reads (MR) reported for all reads mapped to the indicated assembly. Percent total (% Total) is assembly-specific mapped reads relative to input reads. Rows marked with "N/A" or not applicable did not have ERCC synthetic spike-in controls added prior to sequencing.

Virus	Model	Sample	# Replicates	Total Reads	Total mapped reads (MR)			% Total		
					Host	ERCC	Virus	Host	ERCC	Virus
HSV-1	Lytic	MRC-5 Uninf.	4	8.9E+07	8.8E+07	3.5E+05	2.3E+01	98%	0.4%	0%
		MRC-5 12 hpi	4	9.2E+07	4.6E+07	6.9E+05	4.3E+07	50%	0.7%	47%
		MRC-5 24 hpi	2	5.0E+07	1.8E+07	2.1E+05	2.9E+07	36%	0.4%	57%
		RNaseR MRC5 12 hpi	2	6.8E+07	3.9E+07	N/A	2.7E+07	58%	N/A	40%
	Latent	Murine TG Uninf.	4	1.2E+08	1.1E+08	1.9E+05	3.0E+00	91%	0.2%	0.00%
		Murine TG Inf.	4	1.4E+08	1.2E+08	2.1E+05	4.4E+04	91%	0.2%	0.03%
	Explant induced reactivation	Murine TG explant Uninf.	3	8.7E+07	8.2E+07	8.8E+05	0.0E+00	95%	1.0%	0%
		Murine TG explant Inf.	3	8.8E+07	8.3E+07	9.8E+05	5.2E+04	95%	1.1%	0.06%
		Murine TG explant Inf. + ACV	3	8.5E+07	8.0E+07	9.4E+05	5.9E+04	94%	1.1%	0.07%
	Drug enhanced reactivation	Murine TG explant Uninf. +JQ1	3	9.3E+07	8.9E+07	1.1E+06	3.0E+00	95%	1.2%	0.00%
		Murine TG explant Inf. +JQ1	3	9.1E+07	8.6E+07	1.1E+06	6.3E+04	95%	1.2%	0.07%
KSHV	Lytic	LEC Uninf.	2	1.5E+08	1.4E+08	1.7E+06	2.0E+01	98%	1.2%	0.0%
		LEC 3dpi	2	1.5E+08	1.1E+08	1.4E+06	3.2E+07	75%	1.0%	22%
		LEC +RNaseR	2	5.2E+07	4.8E+07	N/A	1.9E+06	93%	N/A	4%
	Pre-Latent	HUV Uninf.	3	2.3E+08	2.2E+08	3.1E+06	9.0E+00	98%	1.3%	0.0%
		HUV 3dpi	3	2.2E+08	2.1E+08	2.7E+06	8.6E+05	97%	1.3%	0.4%
	Lytic reactivation	iSLK-BAC16 Unind.	8	2.6E+08	2.5E+08	1.7E+06	1.5E+05	97%	0.7%	0.06%
		iSLK-BAC16 3dpi	6	1.7E+08	5.3E+07	1.5E+06	1.1E+08	31%	0.9%	63%
MHV68	Lytic	3T3 Uninf.	2	6.6E+07	6.0E+07	1.3E+05	5.2E+01	91%	0.2%	0.0%
		3T3 18 hpi	2	6.9E+07	4.0E+07	2.4E+05	2.3E+07	58%	0.3%	33%
	Latent	Murine GC B-Cell Uninf.	3	1.1E+08	8.6E+07	N/A	1.3E+03	81%	N/A	0.0%
		Murine GC B-Cell Inf.	6	2.5E+08	2.2E+08	N/A	1.4E+05	91%	N/A	0.06%
	Lytic reactivation	HERIT Unind.	2	6.3E+07	5.6E+07	1.4E+05	2.9E+04	90%	0.2%	0.0%
		HERIT 24 hpi	2	7.0E+07	5.8E+07	1.4E+05	3.7E+06	82%	0.2%	5%

Appendix Table S2. High confidence circRNAs in lytic, latent, and reactivation models.

Overview of high confidence circRNA calls made using CHARLIE. All calls required ≥3 BSJ counts to be reported.

Virus	Model	Sample	# Unique BSJ variants		# BSJ MR		Avg Read Count/BSJ		BSJ MR/All MR	
			Host	Virus	Host	Virus	Host	Virus	Host	Virus
HSV-1	Lytic	MRC-5 Uninf.	481	0	3,327	0	6.9	N/A	0.004%	N/A
		MRC-5 12 hpi	924	218	7,501	1,797	8.1	8.2	0.02%	0.004%
		MRC-5 24 hpi	576	223	4,784	2,308	8.3	10.3	0.03%	0.008%
		RNaseR MRC5 12 hpi	14,184	4,318	161,051	31,870	11.4	7.4	0.4%	0.1%
	Latent	Murine TG Uninf.	441	0	4,472	0	10.1	N/A	0.004%	N/A
		Murine TG Inf.	513	0	4,439	0	8.7	N/A	0.004%	N/A
	Explant induced reactivation	Murine TG explant Uninf.	379	0	3,375	0	8.9	N/A	0.004%	N/A
		Murine TG explant Inf.	343	0	2,946	0	8.6	N/A	0.004%	N/A
		Murine TG explant Inf. + ACV	431	0	2,794	0	6.5	N/A	0.003%	N/A
	Drug enhanced reactivation	Murine TG explant Uninf. +JQ1	431	0	4,228	0	9.8	N/A	0.005%	N/A
		Murine TG explant Inf. +JQ1	418	0	3,749	0	9.0	N/A	0.004%	N/A
KSHV	Lytic	LEC Uninf.	3,270	0	24,024	0	7.3	N/A	0.02%	N/A
		LEC 3dpi	2,664	630	18,482	3,200	6.9	5.1	0.02%	0.01%
		LEC +RNaseR	4,334	907	38,944	4,809	9.0	5.3	0.08%	0.3%
	Pre-Latent	HUV Uninf.	2,384	0	48,097	0	20.2	N/A	0.02%	N/A
		HUV 3dpi	5,581	12	50,053	37	9.0	3.1	0.02%	0.004%
	Lytic reactivation	iSLK-BAC16 Unind.	1,458	0	15,447	0	10.6	N/A	0.006%	N/A
		iSLK-BAC16 3dpi	748	1,578	7,294	11,212	9.8	7.1	0.01%	0.01%
MHV68	Lytic	3T3 Uninf.	280	0	1,848	0	6.6	N/A	0.003%	N/A
		3T3 18 hpi	489	489	3,504	2,098	7.2	4.3	0.009%	0.009%
	Latent	Murine GC B-Cell Uninf.	1,474	0	6,728	0	4.6	N/A	0.008%	N/A
		Murine GC B-Cell Inf.	2,883	7	13,798	25	4.8	3.6	0.006%	0.02%
	Lytic reactivation	HERIT Unind.	209	0	1,326	0	6.3	N/A	0.002%	N/A
		HERIT 24 hpi	344	24	2,063	100	6.0	4.2	0.004%	0.003%

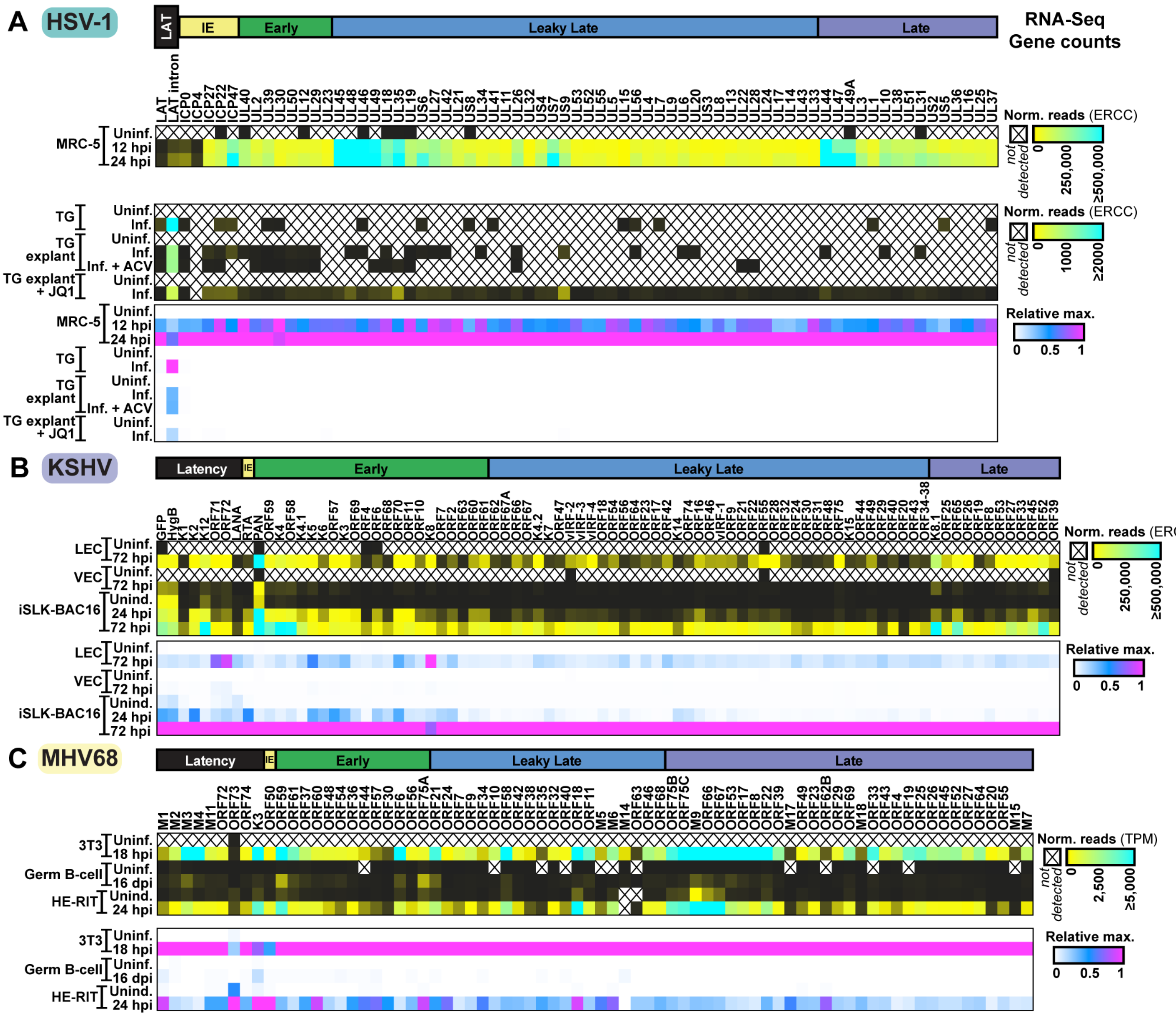

Appendix Figure S1. Viral gene expression in HSV-1, KSHV, and MHV68 models

Viral gene counts for RNA-Seq data in Fig. 1 normalized to ERCC spike-in reads (HSV-1, KSHV) or as transcripts per million, TPM (MHV68). Normalized read values or relative maximum in each column are plotted. Data is the average of biological replicates. Genes are clustered by transcriptional class and labeled as LAT or Latency, IE (immediate early), Early, Leaky Late, Late.

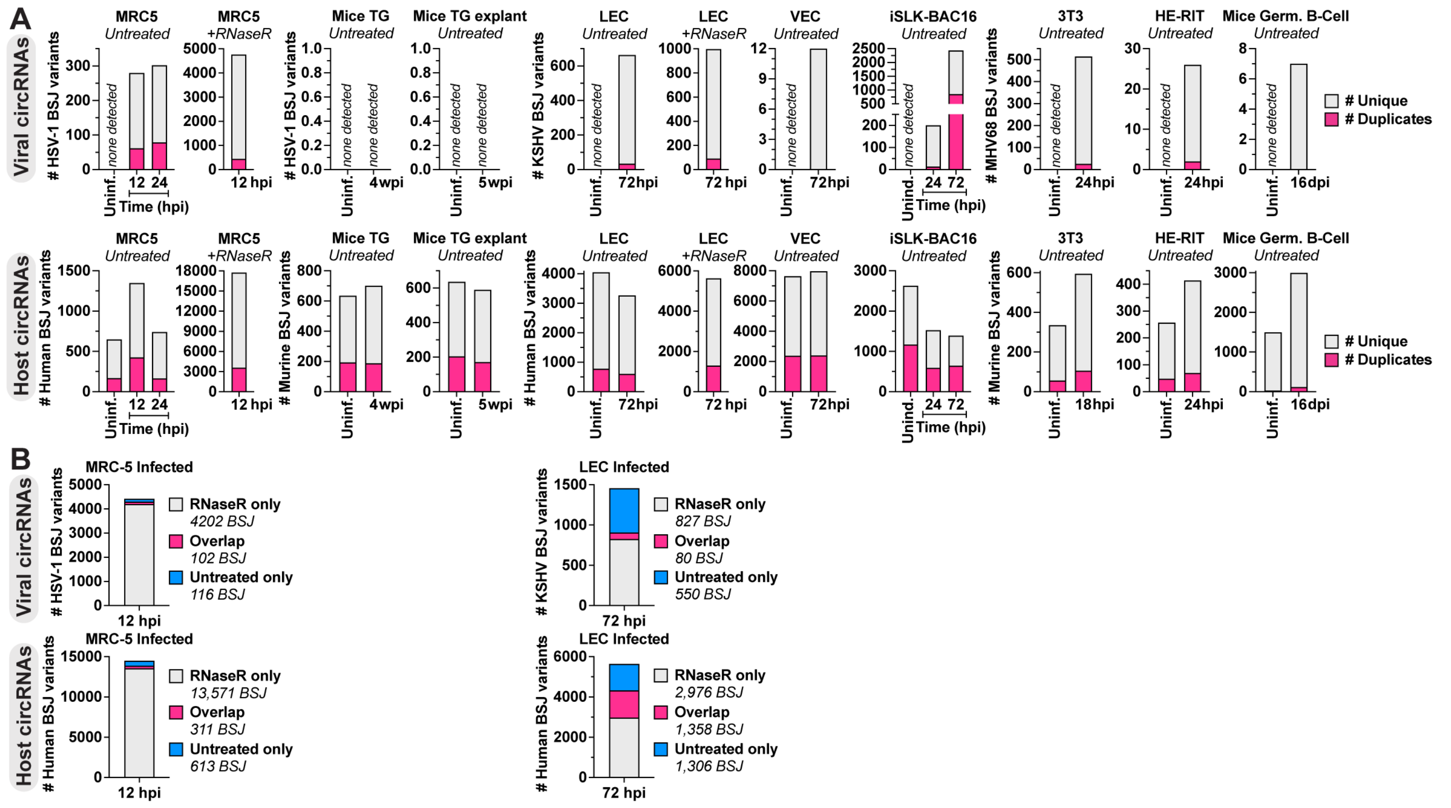

Appendix Figure S2. Reproducibility of high confidence circRNA calls

High confidence viral and host circRNA calls for data in Fig. 1. Overlap was determined using genomic BSJ positions. A) The number of unique or duplicate (relative to other biological replicates) high confidence circRNAs are reported. B) The number of high confidence viral and host BSJ variants found in untreated and/or RNase R treated samples is reported.

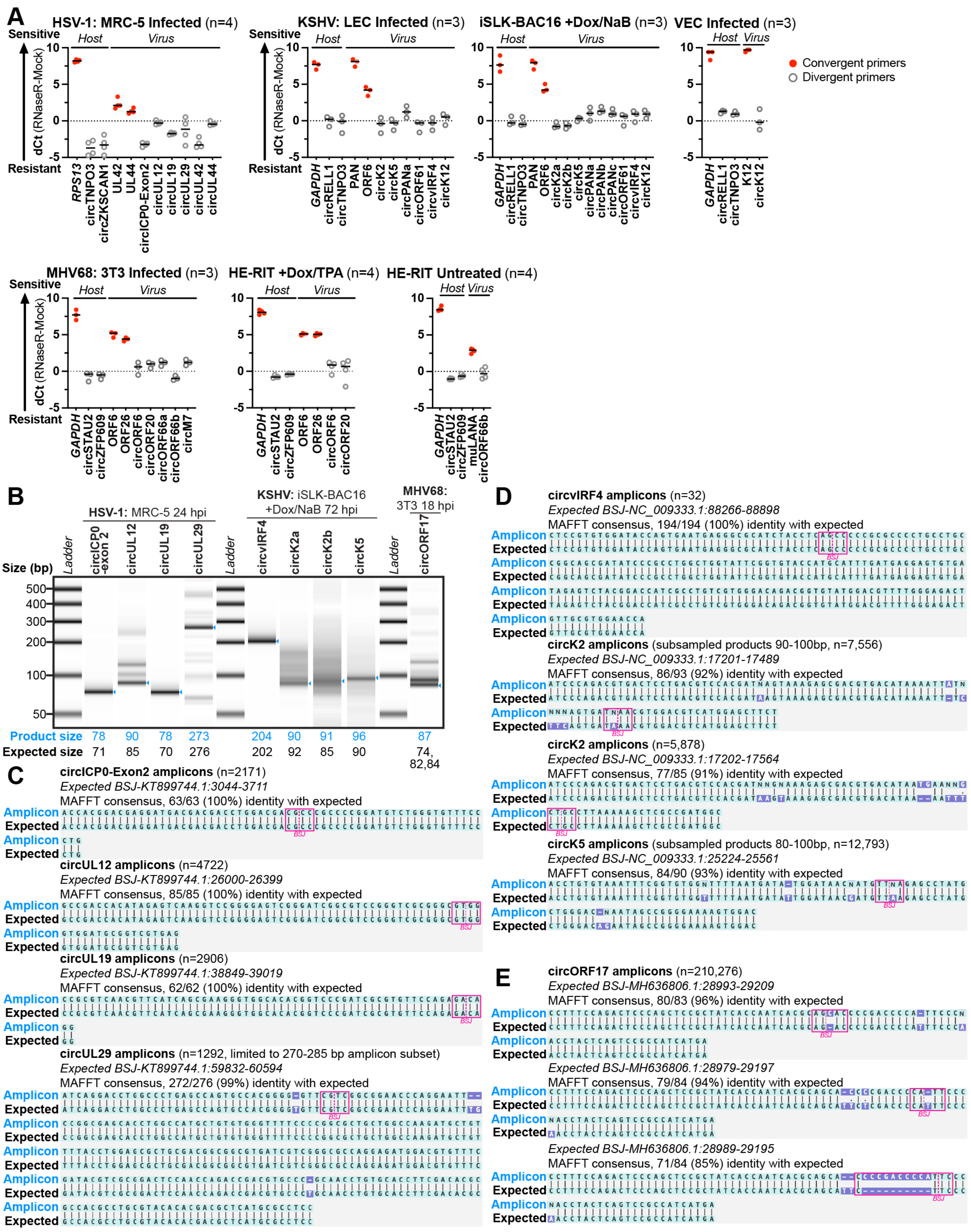
A
HSV-1: MRC-5 Infected (n=4)
KSHV: LEC Infected (n=3)
iSLK-BAC16 +Dox/NaB (n=3)
VEC Infected (n=3)
Convergent primers
Divergent primers
Sensitive
Resistant
dCt (RNaseR-Mock)
Host
Virus
RPS13
circTNPO3
circZKSCAN1
UL42
UL44
circICP0-Exon2
circUL12
circUL19
circUL29
circUL42
circUL44
GAPDH
circRELL1
PAN
ORF6
circK2
circK5
circPANa
circORF61
circvIRF4
circK12
ORF26
circK2a
circK2b
circPANb
circPANc
K12
MHV68: 3T3 Infected (n=3)
HE-RIT +Dox/TPA (n=4)
HE-RIT Untreated (n=4)
circSTAU2
circZFP609
ORF20
circORF6
circORF20
circORF66a
circORF66b
circM7
muLANA
B
HSV-1: MRC-5 24 hpi
KSHV: iSLK-BAC16 +Dox/NaB 72 hpi
MHV68: 3T3 18 hpi
Size (bp)
Ladder
circICP0 -exon 2
circUL12
circUL19
circUL29
circvIRF4
circK2a
circK2b
circK5
circORF17
500
400
300
200
100
50
Product size 78 90 78 273 204 90 91 96 87
Expected size 71 85 70 276 202 92 85 90 74, 82,84
C
circICP0-Exon2 amplicons (n=2171)
Expected BSJ-KT899744.1:3044-3711
MAFFT consensus, 63/63 (100%) identity with expected
circUL12 amplicons (n=4722)
Expected BSJ-KT899744.1:26000-26399
MAFFT consensus, 85/85 (100%) identity with expected
circUL19 amplicons (n=2906)
Expected BSJ-KT899744.1:38849-39019
MAFFT consensus, 62/62 (100%) identity with expected
circUL29 amplicons (n=1292, limited to 270-285 bp amplicon subset)
Expected BSJ-KT899744.1:59832-60594
MAFFT consensus, 272/276 (99%) identity with expected
Amplicon
Expected
BSJ
D
circvIRF4 amplicons (n=32)
Expected BSJ-NC_009333.1:88266-88898
MAFFT consensus, 194/194 (100%) identity with expected
circK2 amplicons (subsampled products 90-100bp, n=7,556)
Expected BSJ-NC_009333.1:17201-17489
MAFFT consensus, 86/93 (92%) identity with expected
circK2 amplicons (n=5,878)
Expected BSJ-NC_009333.1:17202-17564
MAFFT consensus, 77/85 (91%) identity with expected
circK5 amplicons (subsampled products 80-100bp, n=12,793)
Expected BSJ-NC_009333.1:25224-25561
MAFFT consensus, 84/90 (93%) identity with expected
E
circORF17 amplicons (n=210,276)
Expected BSJ-MH636806.1:28993-29209
MAFFT consensus, 80/83 (96%) identity with expected
Expected BSJ-MH636806.1:28979-29197
MAFFT consensus, 79/84 (94%) identity with expected
Expected BSJ-MH636806.1:28989-29195
MAFFT consensus, 71/84 (85%) identity with expected

Appendix Figure S3. Divergent primer validation

A) RNase R protection assay for RNA from models described in Fig. 1. cDNA samples were amplified with divergent (grey) or convergent (red) primers. Values are delta Ct (RNase R - Mock Ct), data points are biological replicates (n=3-4) and horizontal lines are the average. B-E) cDNA from RNase R digested RNA was PCR amplified with divergent primers. Amplicons were B) visualized on a TapeStation or C-E) sequenced using long-read sequencing (Plasmidsaurus). The Multiple Alignment using Fast Fourier Transform (MAFFT) consensus for each amplicon was aligned to the expected BSJ sequence identified by CHARLIE, percent matching is reported.

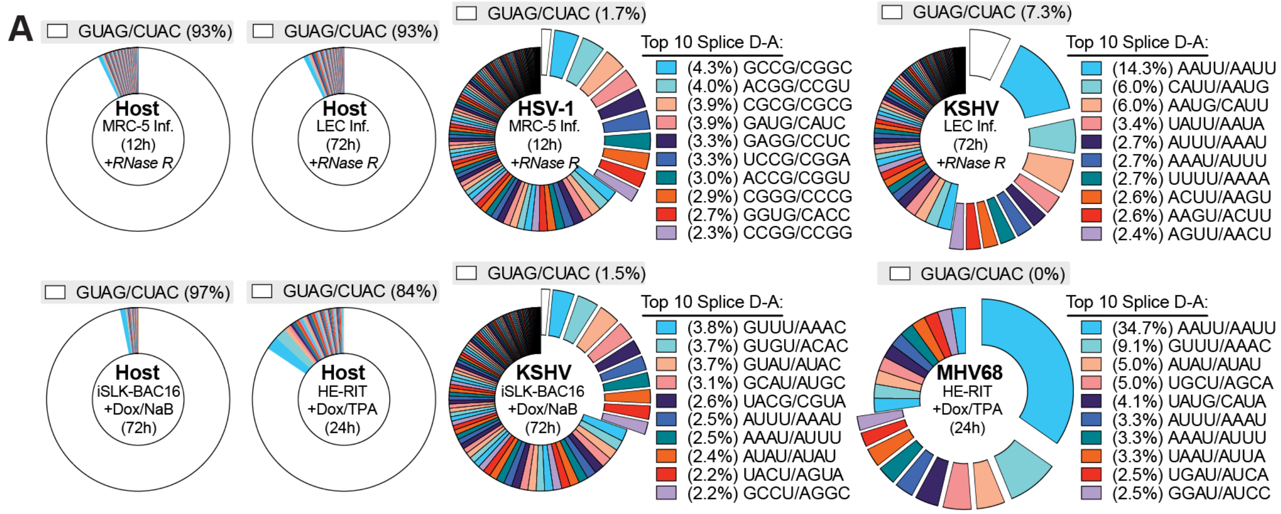

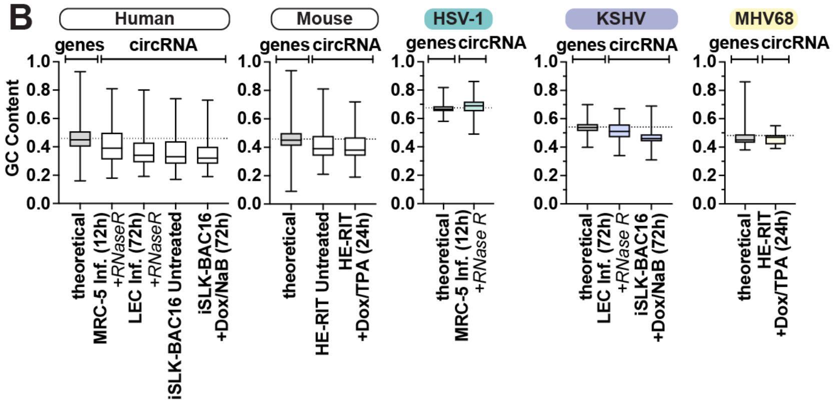

Sample Group			Mean GC content	Discrepancy	P-Value
		Human Genes: theoretical GC usage	*46.1%*	*N/A*	*N/A*
**Human circRNA**	+RNaseR	MRC-5 Inf. (12h)	41.3%	-4.8%	<0.0001
	+RNaseR	LEC Inf. (72h)	37.2%	-8.9%	<0.0001
	N/A	iSLK-BAC16 Untreated	36.4%	-9.7%	<0.0001
	N/A	iSLK-BAC16 +Dox/NaB (72h)	34.8%	-22.3%	<0.0001
		Mouse Genes: theoretical GC usage	*45.8%*	*N/A*	*N/A*
**Mouse circRNA**	N/A	HE-RIT Untreated	41.2%	-4.6%	<0.0001
	N/A	HE-RIT +Dox/TPA (24h)	40.2%	-5.6%	<0.0001
		HSV-1 Genes: theoretical GC usage	*67.4%*	*N/A*	*N/A*
**HSV-1 circRNA**	+RNaseR	MRC-5 Inf. (12h)	68.5%	**1.1%**	<0.0001
		KSHV Genes: theoretical GC usage	*53.8%*	*N/A*	*N/A*
**KSHV circRNA**	+RNaseR	LEC Inf. (72h)	51.3%	-2.5%	<0.0001
	N/A	iSLK-BAC16 +Dox/NaB (72h)	46.4%	-7.4%	<0.0001
		MHV68 Genes: theoretical GC usage	*48.3%*	*N/A*	*N/A*
**MHV68 circRNA**	N/A	HE-RIT +Dox/TPA (24h)	46.1%	-2.2%	<0.0001

Appendix Figure S4. CircRNA cis-element analysis for additional lytic infection models

Cis-element analysis was performed for high confidence circRNAs identified in RNA-Seq data from Fig. 1. If indicated, samples were treated with RNase R prior to RNA-Seq. A) Splice donor-acceptor frequency for circRNAs identified in lytic infection models, cis-elements are reported as sense and antisense sequences. The percentage of the total which use the canonical splice donor acceptor (GU-AG/CU-AC) are reported above. B) GC content of 100 nucleotides flanking BSJ variants relative to the theoretical GC content of genes for an organism. Wilcoxon t-tests were performed, relative to the theoretical gene GC content, to test significance.

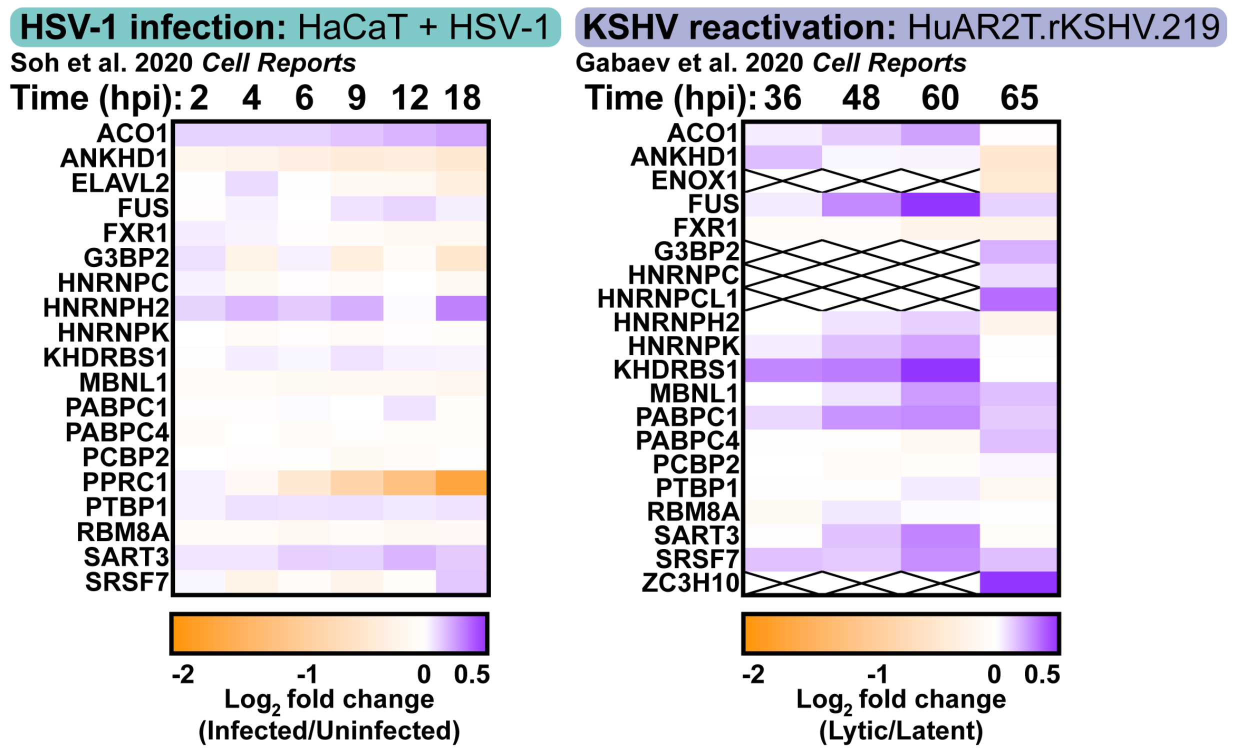

Appendix Figure S5. Protein levels of predicted RBP-circRNA partners during infection

Tandem mass tag mass spectrometry data for predicted RBP partners in Fig. 3E from A) HSV-1 infected HaCaT (human immortalized keratinocytes) (Soh *et al*, 2020) or B) lytic HuAR2T.rKSHV.219 (immortalized human umbilical vein endothelial cells) (Gabaev *et al*, 2020). HSV-1 data is the $\log_2$ fold change of infected/mock-infected samples, values are the average of biological duplicates. KSHV data is the $\log_2$ fold change of cells transduced with a lentiviral vector expressing RTA (lytic) verse cells transduced with a lentiviral vector expressing BFP (latent). 36, 48, and 60 hpi data is from one biological replicate. 65 hpi data is the average of biological duplicates. Squares marked with an “X” did not have any peptides detected for the indicated protein.

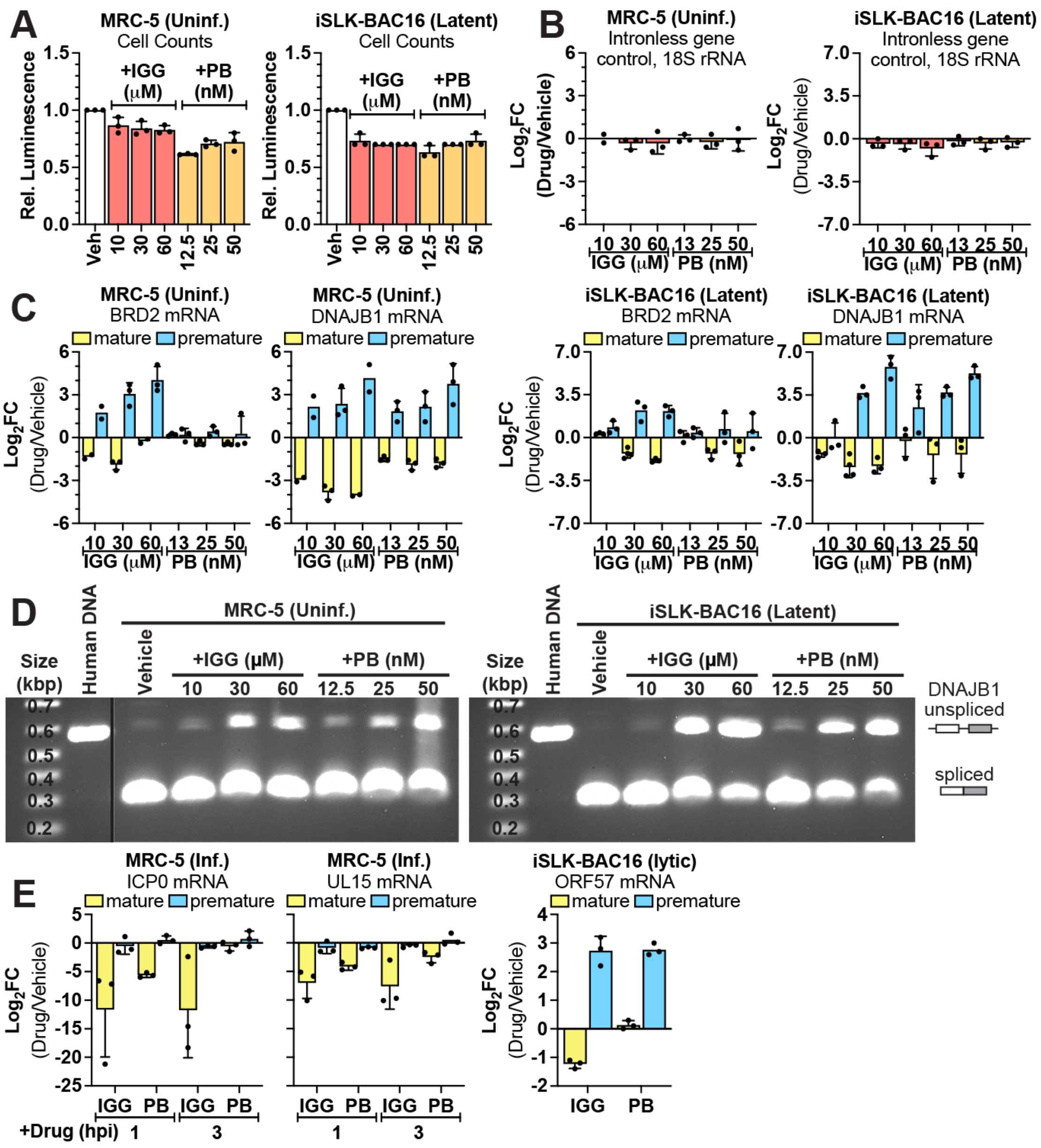

Appendix Figure S6. Impact of spliceosome inhibition on infection models

A-D) 10, 30, 60 µM Isoginkgetin (IGG) or 12.5, 25, 50 nM Pladienolide B (PB) were added for 24 hours to either uninfected MRC-5 or latent iSLK-BAC16. E) HSV-1 infected MRC-5 were treated with 30 µM IGG or 25 nM PB at 1 or 3 hours post infection and collected at 24 hours post infection. iSLK-BAC16 were treated with 30 µM IGG or 25 nM PB and at 24 hours after lytic reactivation (+Dox/NaB) and collected after 24 hours of inhibitor treatment. A) Cell viability was assessed using CellTiterGlo and plotted as luminescence relative to the DMSO or vehicle (Veh) treated control. B, C, E) Transcripts were quantified via qPCR. To detect spliced mRNA, RNA was reverse transcribed using oligo-dT primers, qPCR primers spanned exon-exon junctions, and relative to cDNA qPCR standard curves. To detect unspliced mRNA, RNA was reverse transcribed using random decamers, qPCR primers within intronic regions, and relative to purified genomic DNA standard curves. Data is plotted as the $\log_2$ fold change (inhibitor/vehicle). A-C, E) Data points are biological replicates, column bars are the average, and error bars are standard deviation. D) RNA or purified human genomic DNA was reverse transcribed and PCR amplified using primers which bound upstream of an intron within the DNAJB1 gene. PCR product was run on an agarose gel and stained with ethidium bromide.

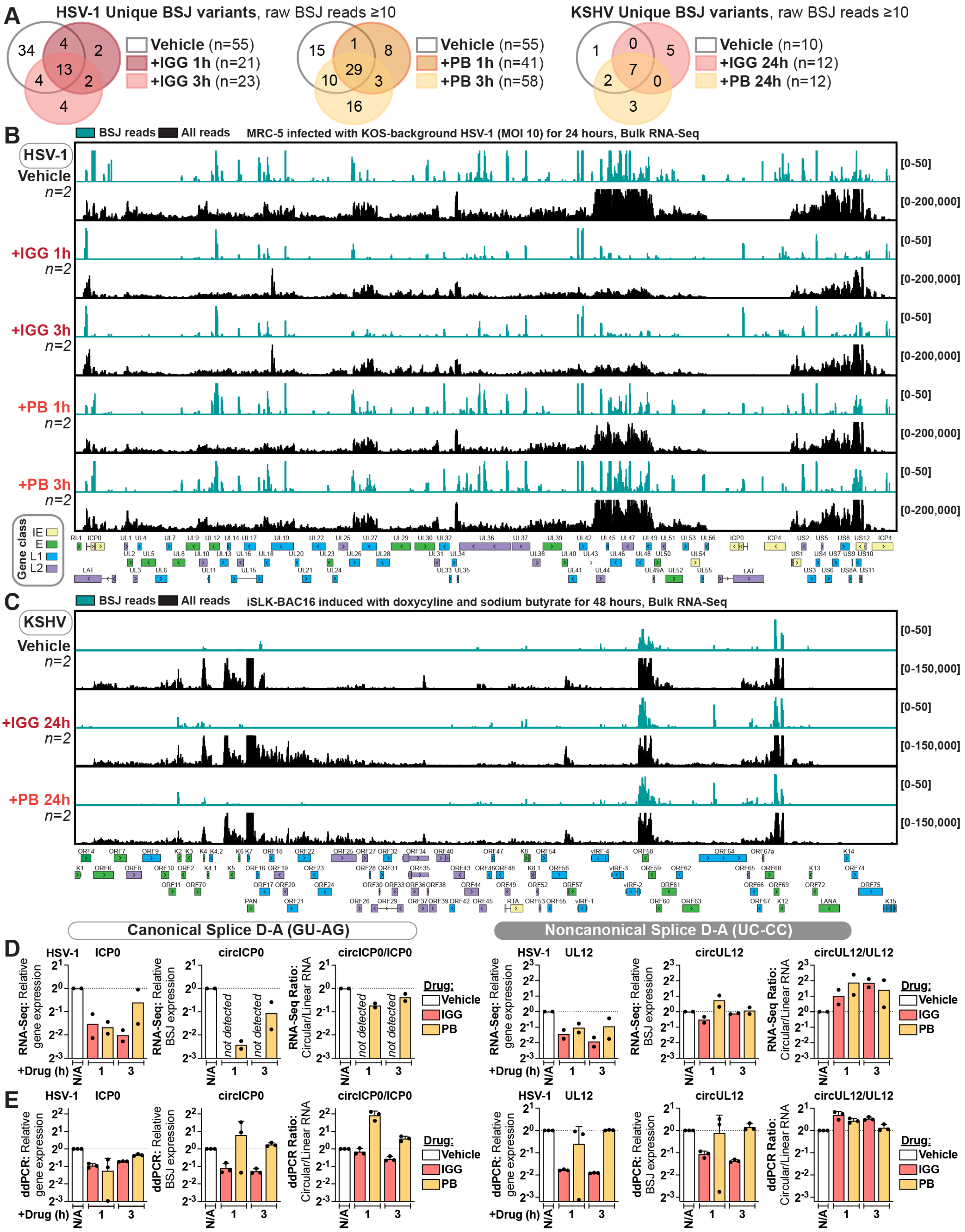
A
HSV-1 Unique BSJ variants, raw BSJ reads ≥10
Vehicle (n=55)
+IGG 1h (n=21)
+IGG 3h (n=23)
+PB 1h (n=41)
+PB 3h (n=58)
KSHV Unique BSJ variants, raw BSJ reads ≥10
Vehicle (n=10)
+IGG 24h (n=12)
+PB 24h (n=12)
B
BSJ reads All reads MRC-5 infected with KOS-background HSV-1 (MOI 10) for 24 hours, Bulk RNA-Seq
HSV-1
Vehicle n=2
+IGG 1h n=2
+IGG 3h n=2
+PB 1h n=2
+PB 3h n=2
[0-50]
[0-200,000]
Gene class IE E L1 L2
C
BSJ reads All reads iSLK-BAC16 induced with doxycyline and sodium butyrate for 48 hours, Bulk RNA-Seq
KSHV
Vehicle n=2
+IGG 24h n=2
+PB 24h n=2
[0-50]
[0-150,000]
Canonical Splice D-A (GU-AG)
Noncanonical Splice D-A (UC-CC)
D
HSV-1 ICP0
circICP0
circICP0/ICP0
HSV-1 UL12
circUL12
circUL12/UL12
RNA-Seq: Relative gene expression
RNA-Seq: Relative BSJ expression
RNA-Seq Ratio: Circular/Linear RNA
not detected
+Drug (h) N/A 1 3
Drug: Vehicle IGG PB
E
ddPCR: Relative gene expression
ddPCR: Relative BSJ expression
ddPCR Ratio: Circular/Linear RNA
+Drug (h) N/A 1 3
Drug: Vehicle IGG PB

Supplementary Figure S7. Quantitation of viral RNAs after spliceosome inhibition.
Data from infection models in Fig. 4. A) Overlapping identity of high-confidence viral circRNAs identified by CHARLIE. Only BSJ with ≥10 raw reads in a given sample were included in the comparison. B-C) Visualization of high confidence HSV-1 and KSHV circRNAs. Green and black traces include circular (back spliced reads) and linear (non-chimeric) reads, respectively. Traces are the sum of raw BSJ or linear read values for biological duplicates. Y-axis minimum and maximum values are shown on the right. Viral genes are shown below and labeled by gene class as IE (yellow), E (green), L1 (blue), and L2 (purple). D) RNA-Seq quantitation of viral circRNAs via CHARLIE, all data is normalized to ERCC spike-in controls and the average of biological duplicates. Transcript ratios were determined by plotting gene expression for a given loci, with circular (BSJ containing reads) over linear (non-chimeric) reads. D) ddPCR quantitation using divergent (circRNA) or convergent (gene) primers (n=3). D-E) Data is plotted relative to a matched vehicle control, with data points being biological replicates, column bars are the average, and error bars are standard deviation.

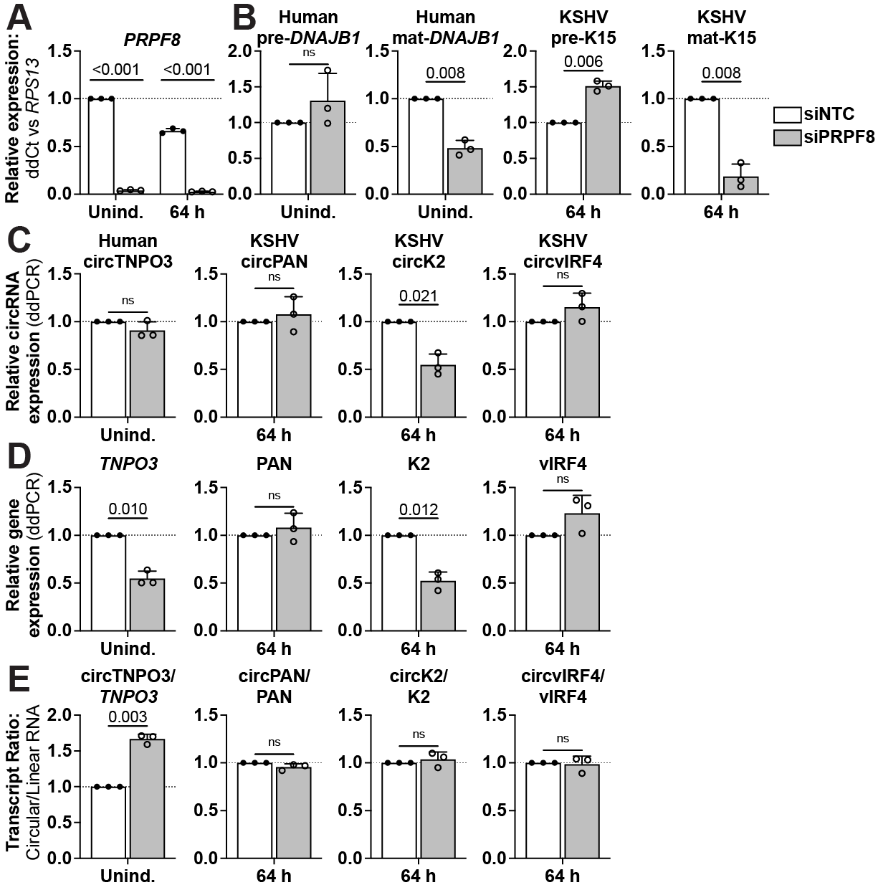

Appendix Figure S8. Impact of spliceosome depletion on KSHV circRNA levels.
iSLK-BAC16 were transfected with siRNAs targeting *PRPF8* or a nontargeting control (NTC) for 8 hours. Subsequently cells were treated with vehicle (Unind.) or Dox and NaB (64 h) for 64 hours. RNA was collected at 72 hours after addition of siRNAs. Transcripts were quantified by A-B) qPCR or C-E) digital droplet PCR. A-B) qPCR data was analyzed as ddCt relative to the reference gene, *RPS13*. C-E) ddPCR quantitation using divergent (circRNA) or convergent (gene) primers (n=3). A-E) All data is plotted relative to a matched siNTC control, with data points being biological replicates (n=3), column bars are the average, and error bars are standard deviation. Two-tailed paired t-tests were performed, all p-values <0.05 are listed.

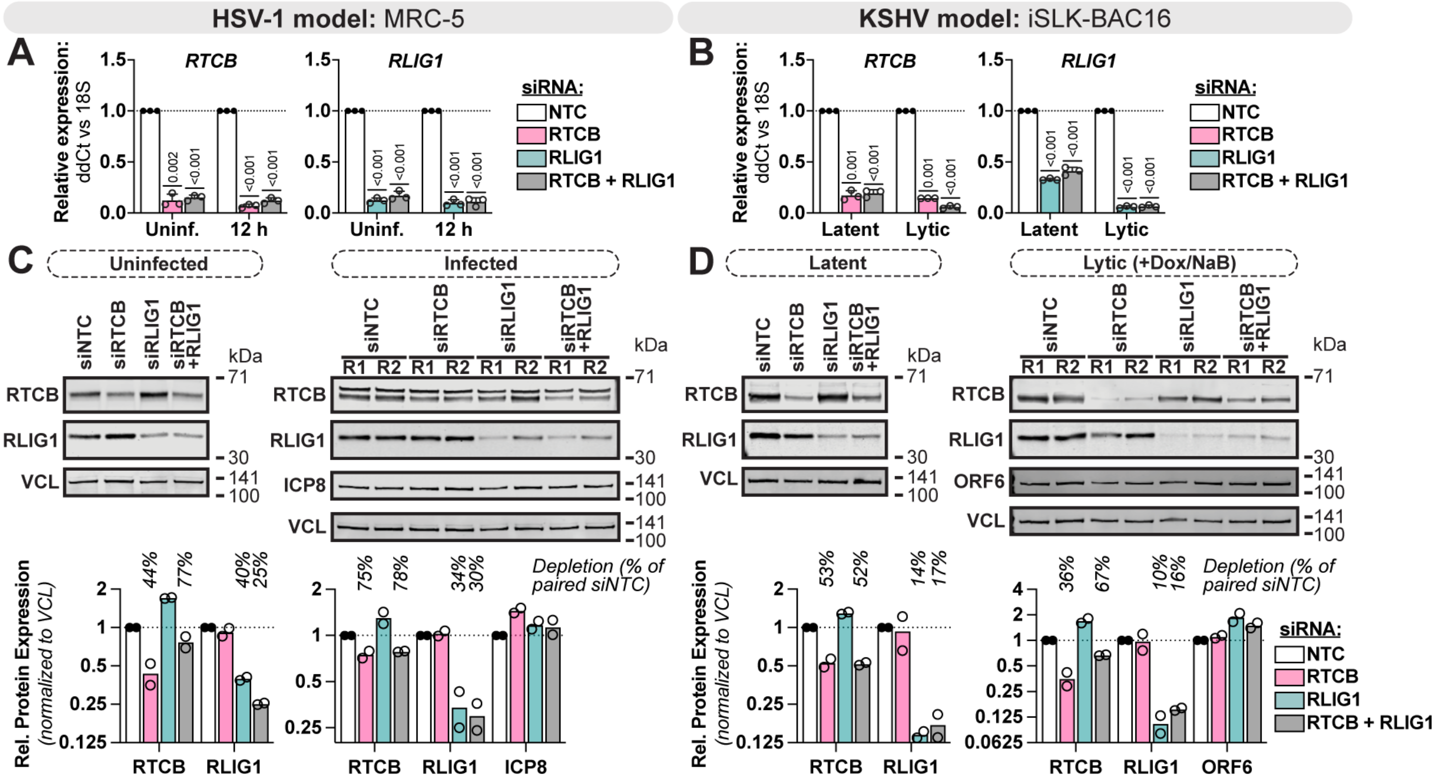

Appendix Figure S9. RNA ligase expression following siRNA depletion

A, C) MRC-5 were transfected with siRNAs targeting RNA ligases (RTCB, RLIG1) or a nontargeting control (NTC) for 2 days. MRC-5 were mock (Uninf.) or HSV-1 infected (strain KOS, MOI 10 PFU/cell) for an additional 12 hours. B, D) iSLK-BAC16 were transfected with siRNAs targeting RNA ligases (RTCB, RLIG1) or a nontargeting control for 24 hours. Subsequently cells were treated with vehicle (Latent) or Dox and NaB for 72 hours (Lytic). A-B) Transcripts quantified by qPCR relative to the reference gene, 18S rRNA. Data points are biological replicates (n=3), column bars are the average, and error bars are standard deviation. All data is relative a paired siNTC sample. Two-tailed paired t-tests were performed. C-D) Protein expression was assessed by immunoblotting and quantified relative to a loading control (VCL). Data points are biological replicates (n=2), column bars are the average, the percentage relative to siNTC is labeled in italics above samples expected to have depletion.

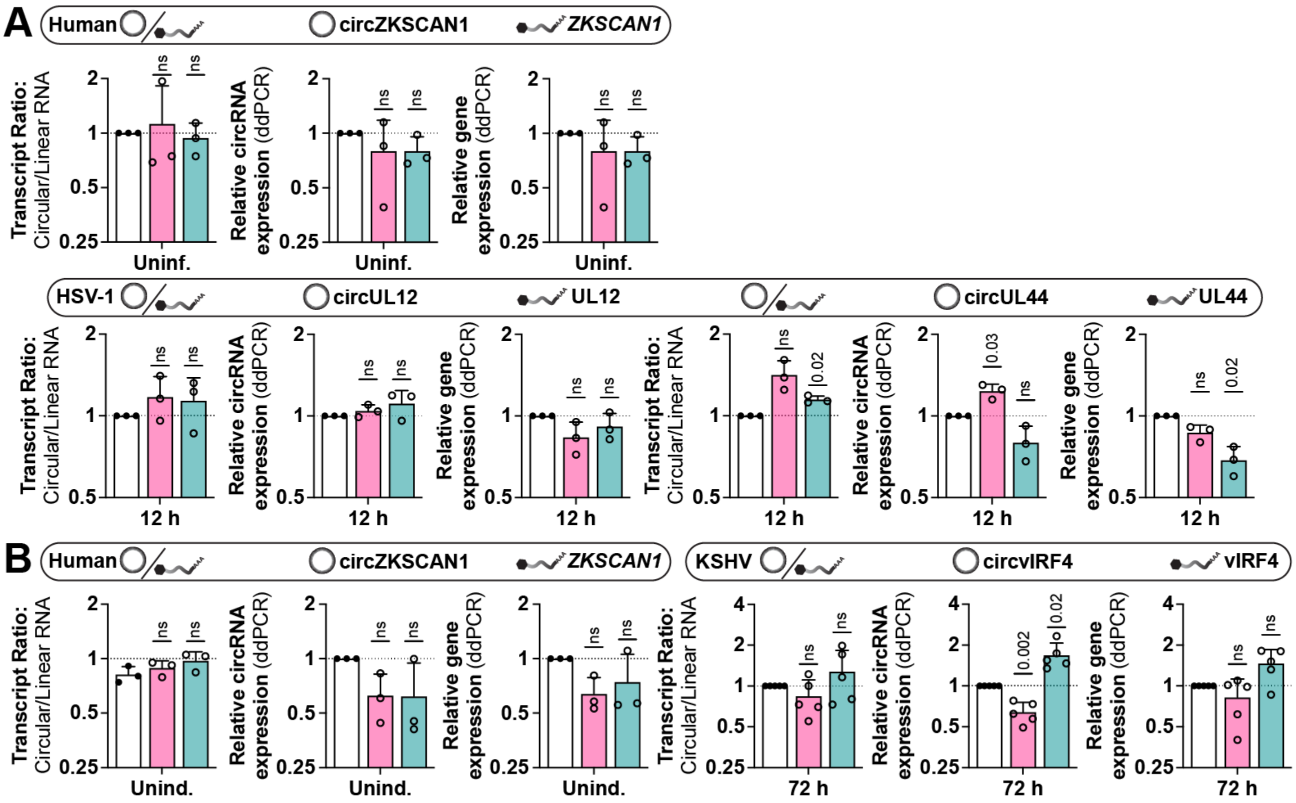

Appendix Figure S10. circRNAs unaffected by RNA ligase depletion

A) MRC-5 were transfected with siRNAs targeting RNA ligases (*RLIG1, RTCB*) or a nontargeting control (NTC) for 48 hours. MRC-5 were mock-infected (Uninf.) or infected with HSV-1 for an additional 12 (12h). iSLK-BAC16 were siRNA transfected for 24 hours followed by mock-treatment (Unind.) or Dox/NaB for 72 hours (72 h). A-B) Transcript ratios quantified by ddPCR using divergent (circular) and convergent (linear) primers. Data points are biological replicates (n=3-5), column bars are the average, and error bars are standard deviation. All data is relative a paired siNTC sample. Paired t-tests were performed, all p-values <0.05 are listed.

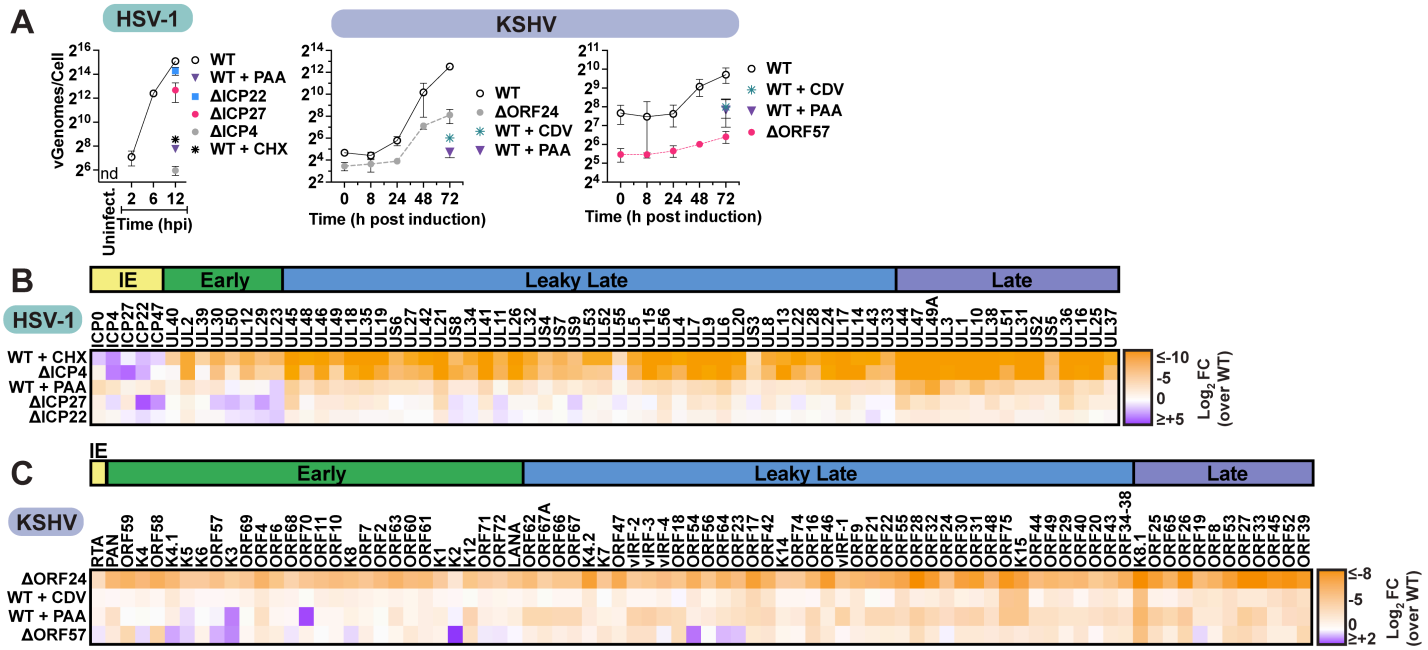

Appendix Figure S11. HSV-1 and KSHV gene expression in mutant viral models

A) qPCR assessment of HSV-1 (n=3) or KSHV (n=2) genome quantity, plotted as the number of viral genomes per cell (vGenomes/Cell). Data points are the average and error bars are standard deviation. B-C) Viral gene expression for RNA-Seq data in Fig. 5 normalized to ERCC spike-in controls, plotted as Log_2FC relative to a paired wildtype. Data is the average of biological duplicates. Genes are clustered by transcriptional class and labeled as LAT (latent), IE (immediate early), Early, Leaky Late (L1), Late (L2).

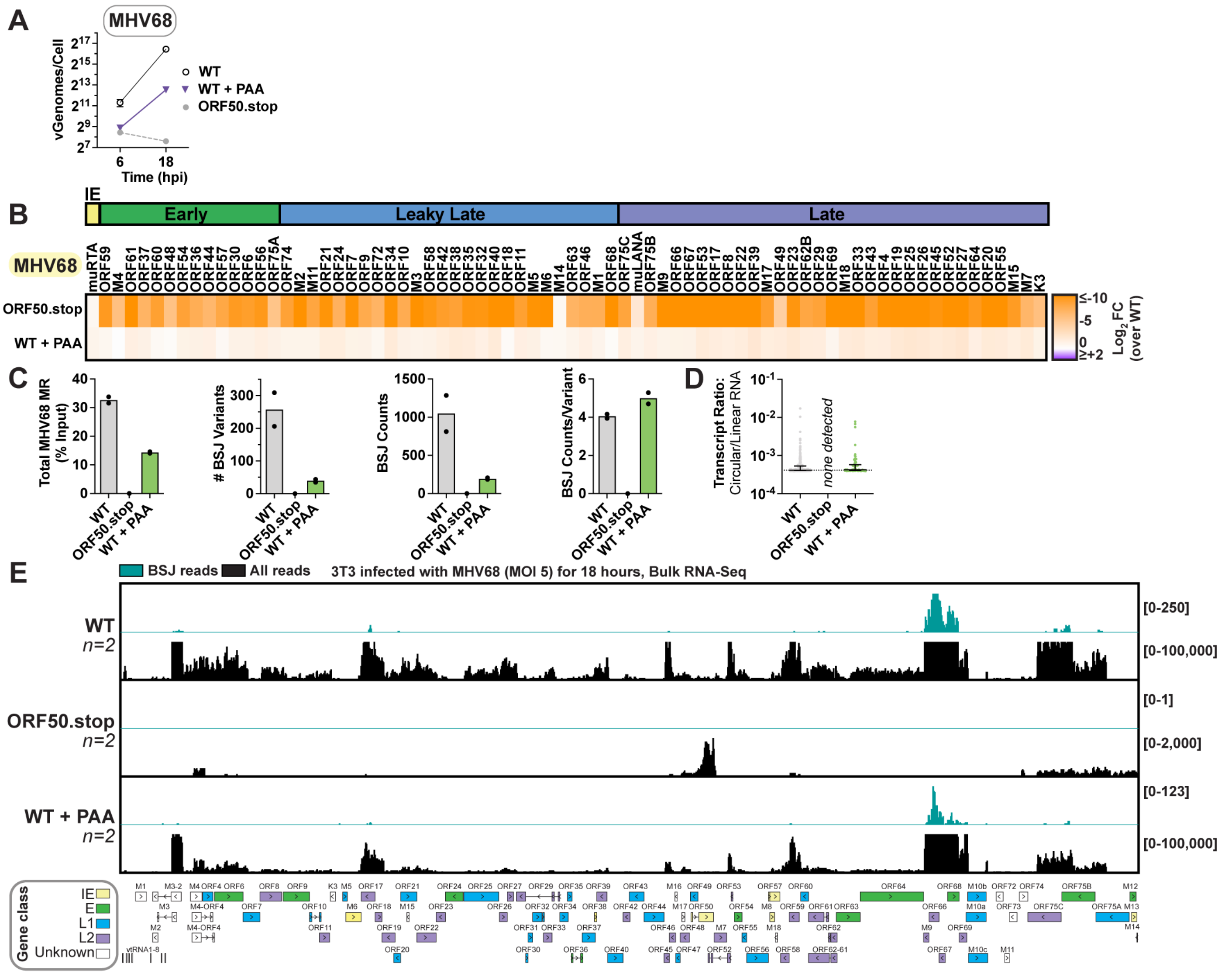

Appendix Figure S12. MHV68 gene expression in mutant viral models

3T3 infected with wildtype MHV68 or ORF50.stop virus at an MOI of 5 PFU/cell. If indicated, 100 µg/mL phosphonoacetic acid (PAA) was added at 1.5 hours post infection. A) qPCR assessment of genome quantity (n=3), plotted as the number of viral genomes per cell (vGenomes/Cell). Data points are the average and error bars are standard deviation. B-D) RNA was collected at 18 hours and RNA-Seq performed. Gene counts were quantified using RNA STAR and normalized to ERCC spike-in reads. BSJ counts and Circular/Linear ratios were quantified using CHARLIE. B) Viral gene expression plotted as Log_2FC relative to a paired wildtype. Data is the average of biological duplicates. Genes are clustered by transcriptional class and labeled as IE (immediate early), Early, Delayed Early, and Late. C) Sequencing overview for all viral mapped reads (MR), as percent total reads (% Total). The number of unique BSJ variants, total BSJ counts, or BSJ counts/variant is reported for high confidence viral circRNAs. Each point is a biological replicate, column bars are the average. D) Circ/Linear ratios for high confidence viral BSJ variants. Each dot represents a unique BSJ, cross-bars are the geometric mean and error bars are the geometric standard deviation. E) Visualization of high confidence circRNAs for MHV68. Blue and black traces include circular (back spliced reads) and linear (non-chimeric) reads, respectively. Traces are the sum of raw BSJ or linear read values for all biological replicates. Y-axis minimum and maximum values are shown on the right. Viral genes are shown below and labeled by gene class as IE (yellow), E (green), L1 (blue), L2 (purple), and unknown (white).

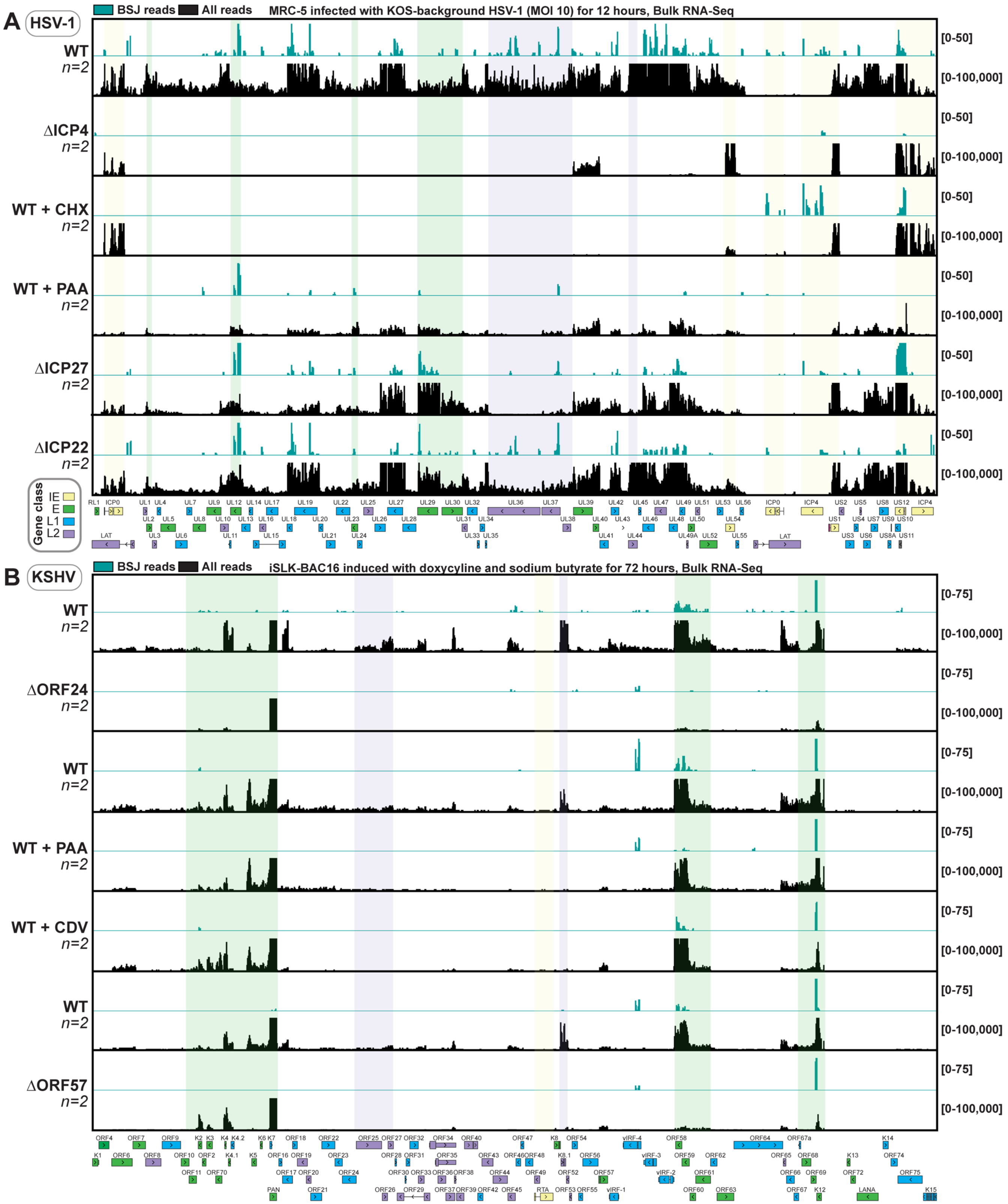

Appendix Figure S13. HSV-1 and KSHV transcript profiles in mutant viral models

Visualization of high confidence HSV-1 and KSHV circRNAs. Blue and black traces include circular (back spliced reads) and linear (non-chimeric) reads. Traces are the sum of raw BSJ or linear read values for biological duplicates. Y-axis minimum and maximum values are shown on the right. Viral genes are shown below and labeled by gene class as IE (yellow), E (green), L1 (blue), and L2 (purple).

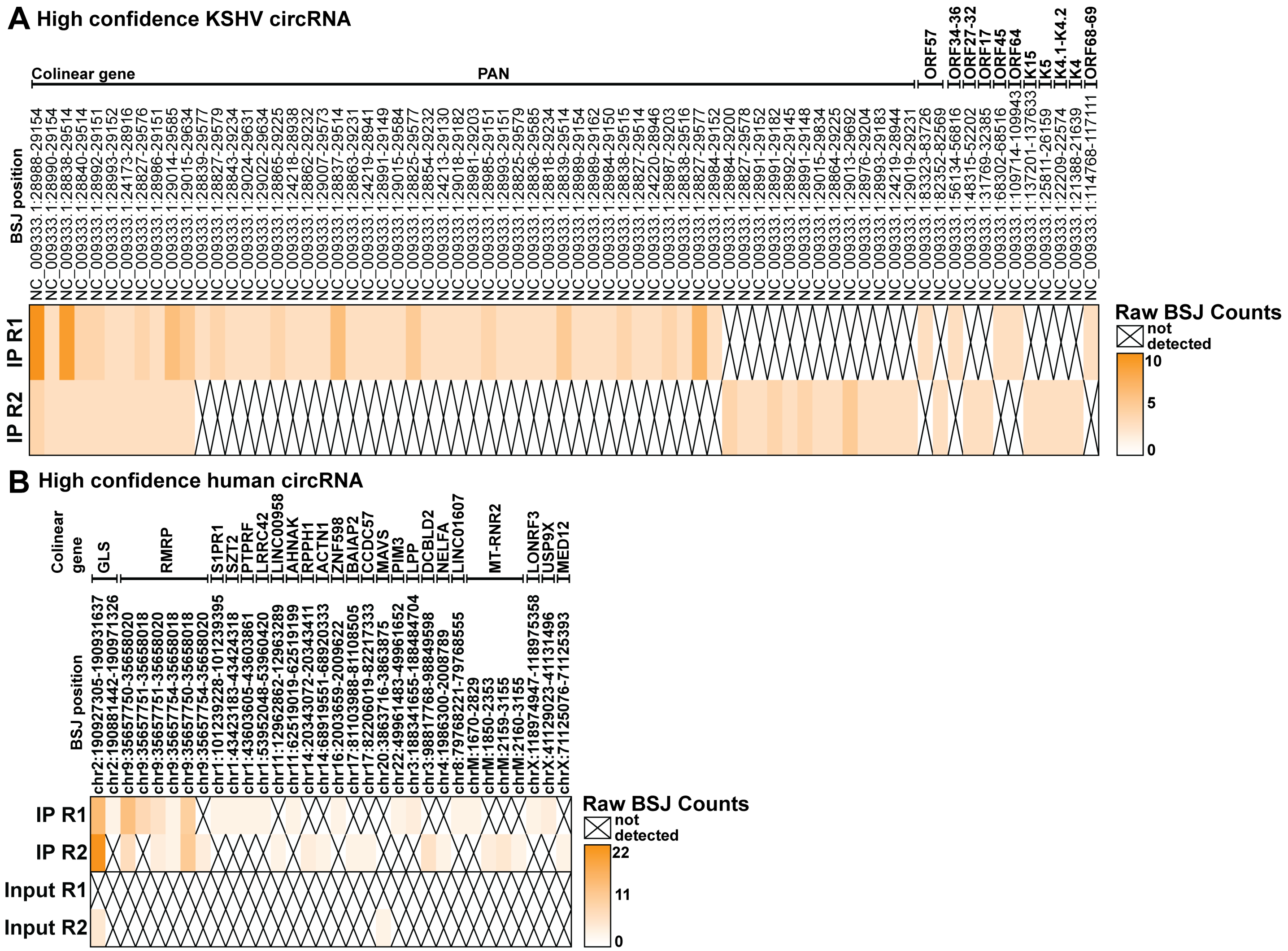

Appendix Figure S14. ORF57 eCLIP heatmap

A-B) ORF57 eCLIP (n=2) was performed on iSLK-BAC16 treated with sodium butyrate and doxycycline for 24 hours. “Input” is a paired, size-selected RNA-Seq wherein ORF57 immunoprecipitation was not performed. CircRNA were quantified using CHARLIE and plotted as raw BSJ counts. BSJ position is listed with colinear genes labeled above. Heatmaps include all high confidence A) viral and B) host circRNAs in the dataset.

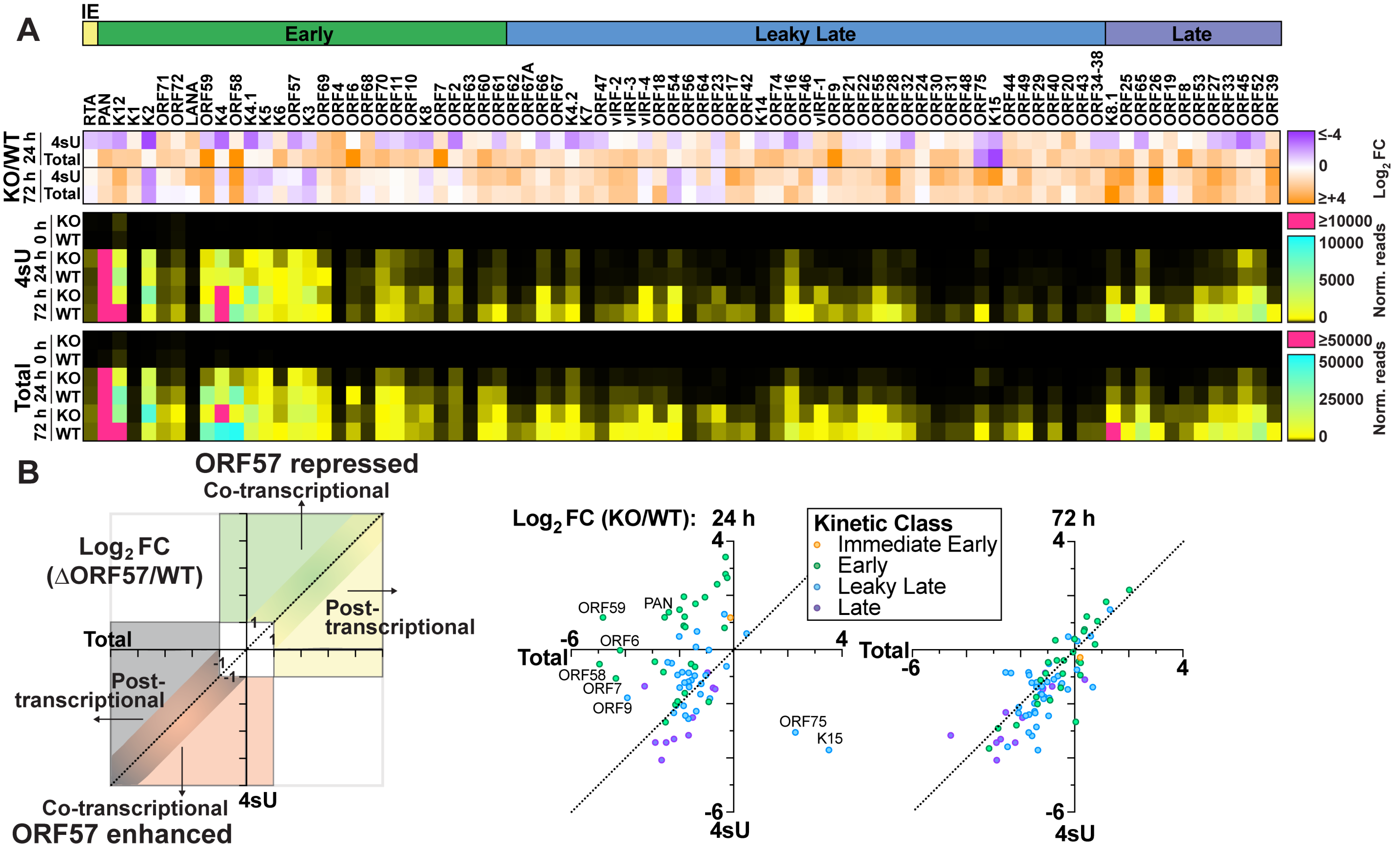

Appendix Figure S15. ORF57 regulation of non-chimeric viral transcripts

Bulk (total) and 4-thiouridine (4sU) RNA-Seq for ΔORF57 (KO) and wildtype (WT) iSLK-BAC16 during lytic reactivation. iSLK-BAC16 were treated with (24 and 72 h) or without (0 h) 1 mM sodium butyrate and 1 µg/mL doxycycline. Gene counts were quantified using RNA STAR and normalized for 4sU and total RNA-Seq as Reads Per Kilobase per Million mapped reads (RPKM) or relative to ERCC spike-ins, respectively. Data is the average of biological duplicates. A) Viral gene expression plotted as normalized reads (norm. reads) or ΔORF57 relative to a paired wildtype control ($\log_2$FC). Genes are clustered by transcriptional class and labeled as IE (immediate early), Early, Leaky Late, and Late. B) Viral gene expression from 24 or 72 hours post induction plotted as $\log_2$FC (KO/WT), where the x-axis is total RNA-Seq data and the y-axis is 4sU-Seq data.

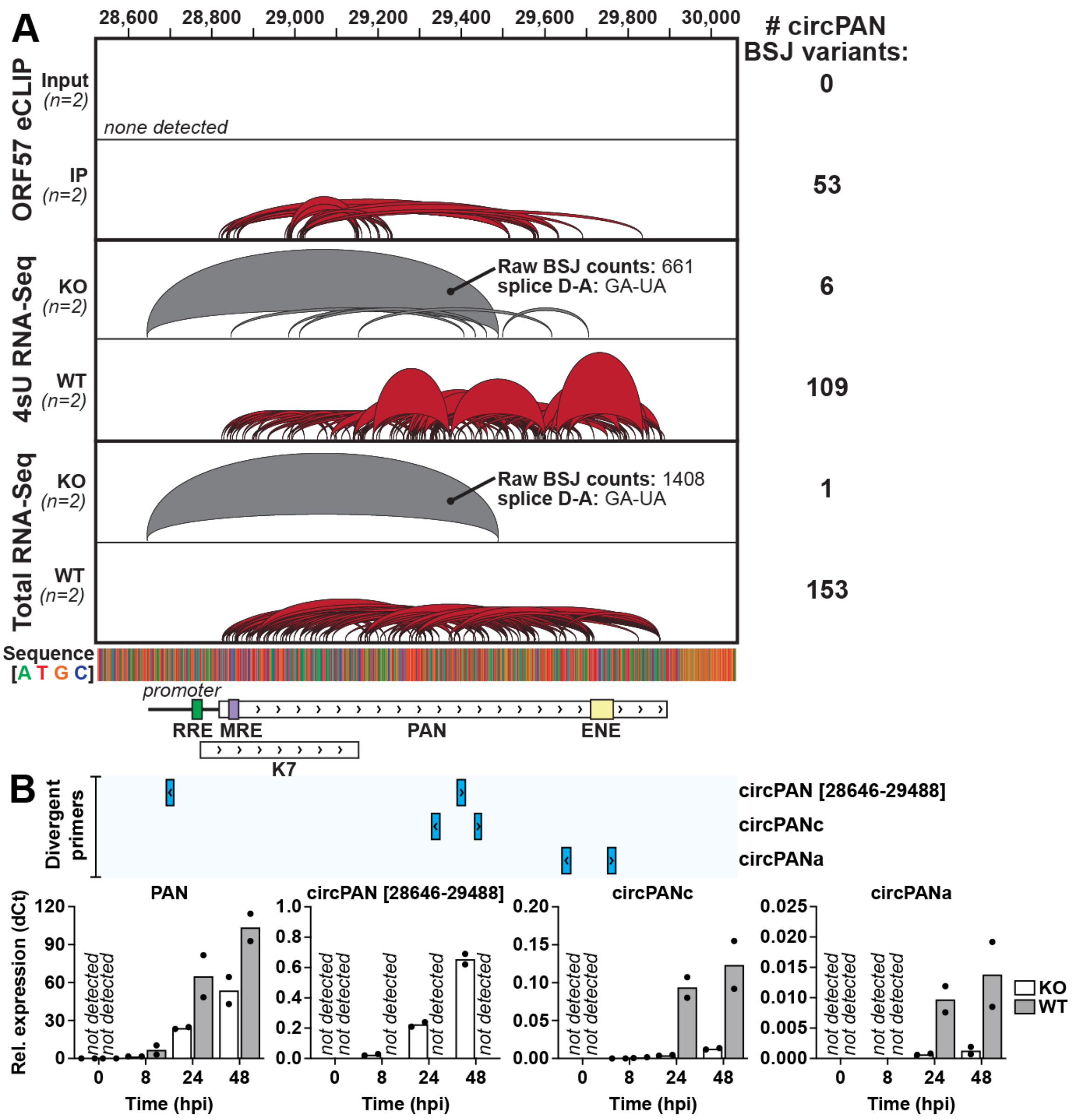

Appendix Figure S16. ORF57 dependence of PAN BSJ variants

A) Visualization of high confidence circRNAs at the PAN locus. ORF57 eCLIP (n=2) was performed on induced iSLK-BAC16 (24 h). "Input" is a paired, size-selected RNA-Seq wherein ORF57 immunoprecipitation was not performed. Total and 4sU RNA-Seq (n=2) was performed on ΔORF57 (KO) or iSLK-BAC16 (WT) cells with (24 h) or without (Unind.) reactivation. Sashimi plots show high confidence circRNA with arcs proportional to raw BSJ counts. Each dataset is the sum of biological duplicates. Viral genes and domains including RTA response element (RRE), MTA response element (MRE), expression and nuclear retention element (ENE) are shown below. B) RNA was collected from ΔORF57 (KO) or iSLK-BAC16 (WT) cells reactivated with doxycline and sodium butyrate for 0, 8, 24, and 48 hours. PAN and circPAN transcripts were measured using qPCR and convergent or divergent primers, respectively. The genomic position of divergent primer sets is shown relative to the PAN locus. Data is plotted as relative expression: $2^{-1\times(Experimental\ gene\ Ct - Reference\ gene\ Ct)}$, using 18S rRNA as the reference gene. Individual data points are biological replicates, column bars are the average.
