# Supplementary material for: Noncanonical circRNA biogenesis driven by alpha and gamma herpesviruses
Source: EMBO J. 2025 Mar 3;44(8):2323–52. doi: 10.1038/s44318-025-00398-0 (PMC12000468; doi:10.1038/s44318-025-00398-0)
Supplement: Supplementary file 8 — Expanded View Figures [file 44318_2025_398_MOESM8_ESM.pdf]

Expanded View Figures

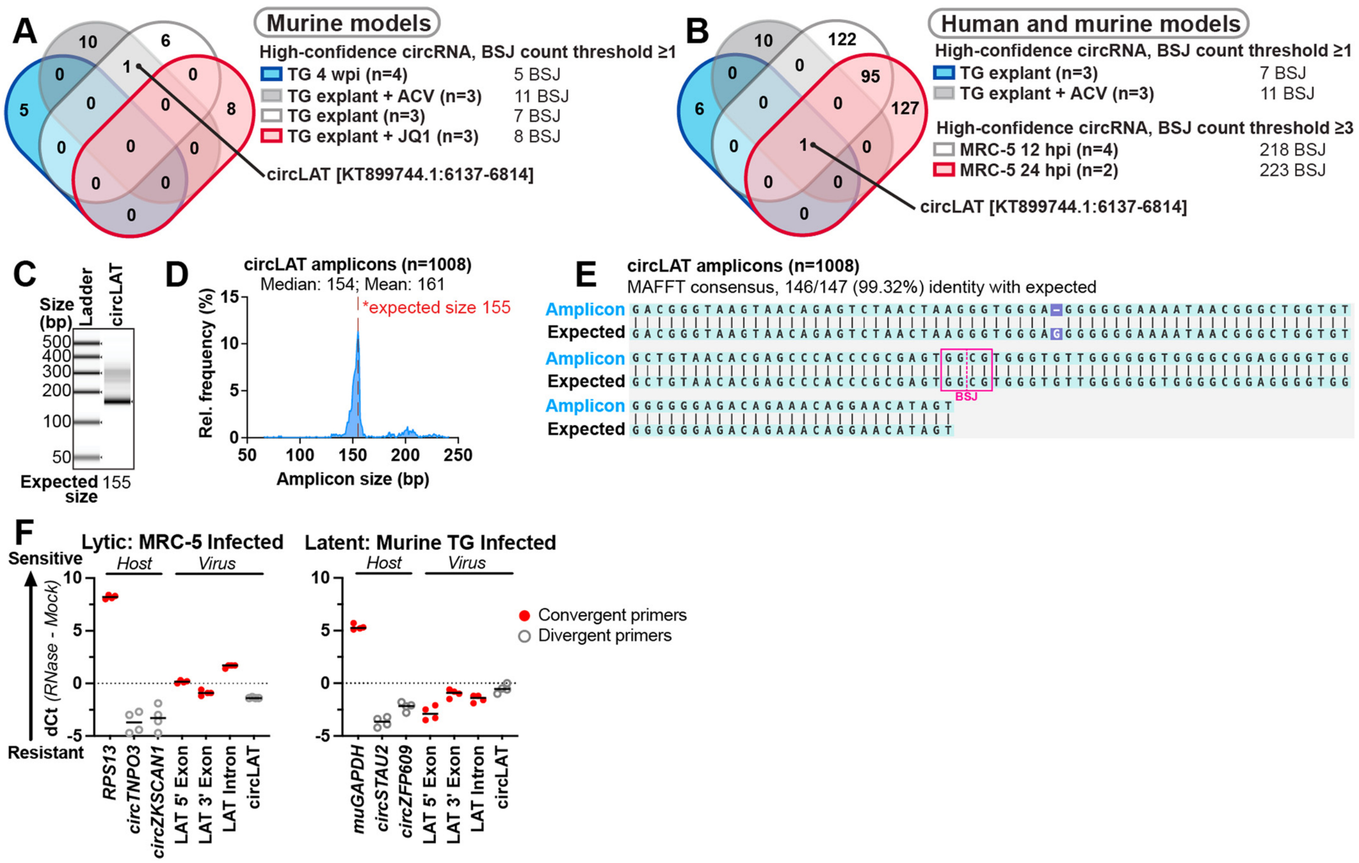

Figure EV1. CircRNA derived from HSV-1 latency-associated transcript.

(**A**, **B**) Our high-confidence circRNA threshold was lowered (count of ≥1 rather than ≥3) to reanalyze murine HSV-1 infection models. The overlapping incidence of these circRNAs is demonstrated for murine and human models. (**C–E**) RNase R-digested RNA (MRC-5 12 hpi) was reverse transcribed and PCR amplified with divergent primers. Amplicons were assessed via electrophoresis (TapeStation) or long-read sequencing (Oxford Nanopore Technology). The MAFFT consensus for circLAT was aligned to the expected BSJ sequence identified by CHARLIE, percent matching is reported. (**F**) RNase R protection assay for RNA from infected MRC-5 (12 hpi) or infected murine TG (4 wpi). cDNA samples were amplified with divergent (gray) or convergent (red) primers. Values are delta Ct (RNase R - Mock Ct), data points are biological replicates ($n = 4$) and horizontal lines are the average.

 © The Author(s)

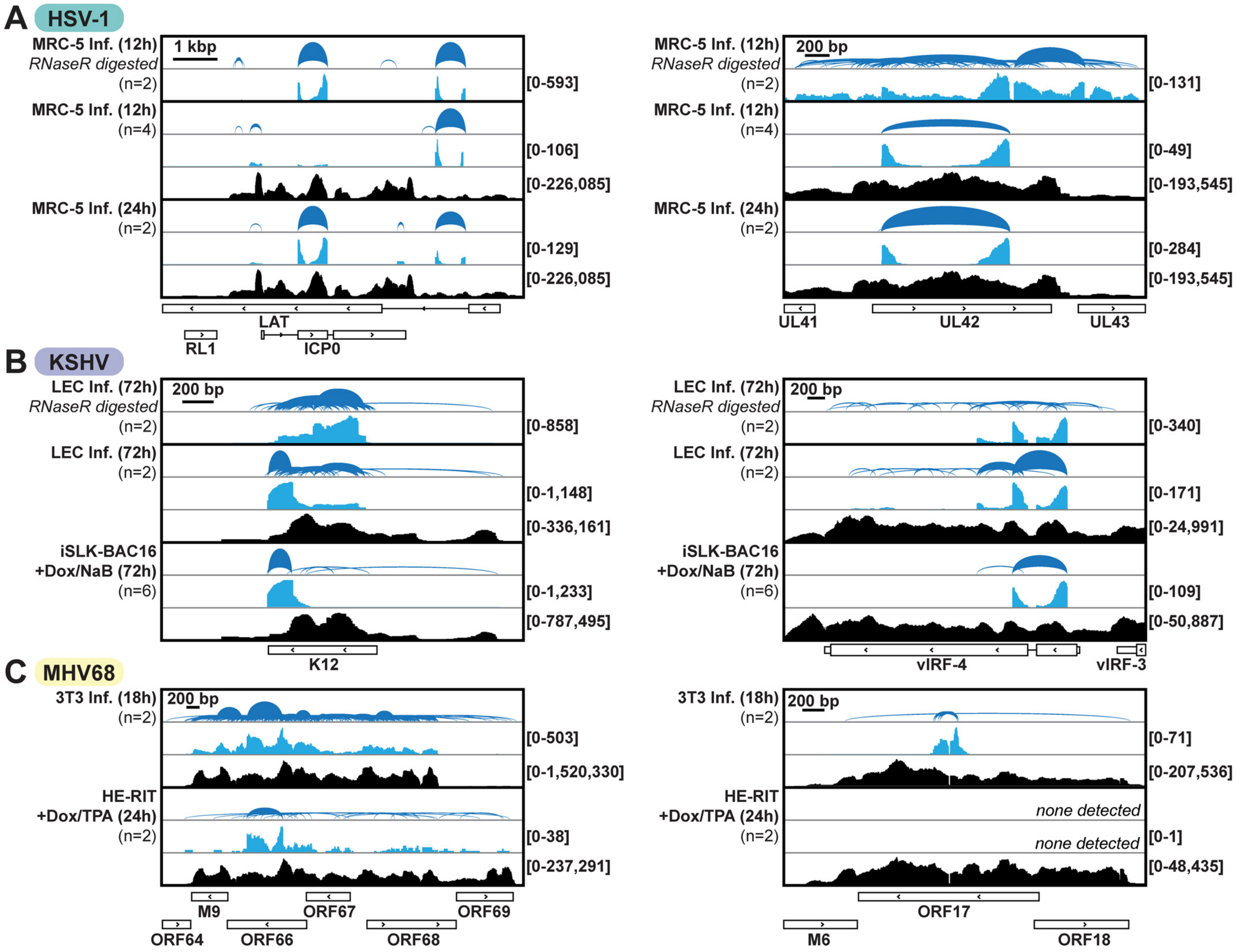

Figure EV2. Prominent examples of herpesvirus circRNAs.

(**A–C**) Visualization of high-confidence circRNAs for HSV-1, KSHV, and MHV68 in lytic models described in Fig. 1. If indicated, RNA samples were treated with RNase R. Viral genes are shown below. Data Information: RNA-Seq was performed and high-confidence circRNAs were called using CHARLIE. Sashimi plots show high-confidence circRNA with arcs proportional to raw BSJ counts. Blue and black traces include circular (back-spliced reads) and linear (non-chimeric) reads, respectively. Traces are the sum of raw BSJ or linear read values for all biological replicates. *Y* axis minimum and maximum values are shown on the right.

© The Author(s)

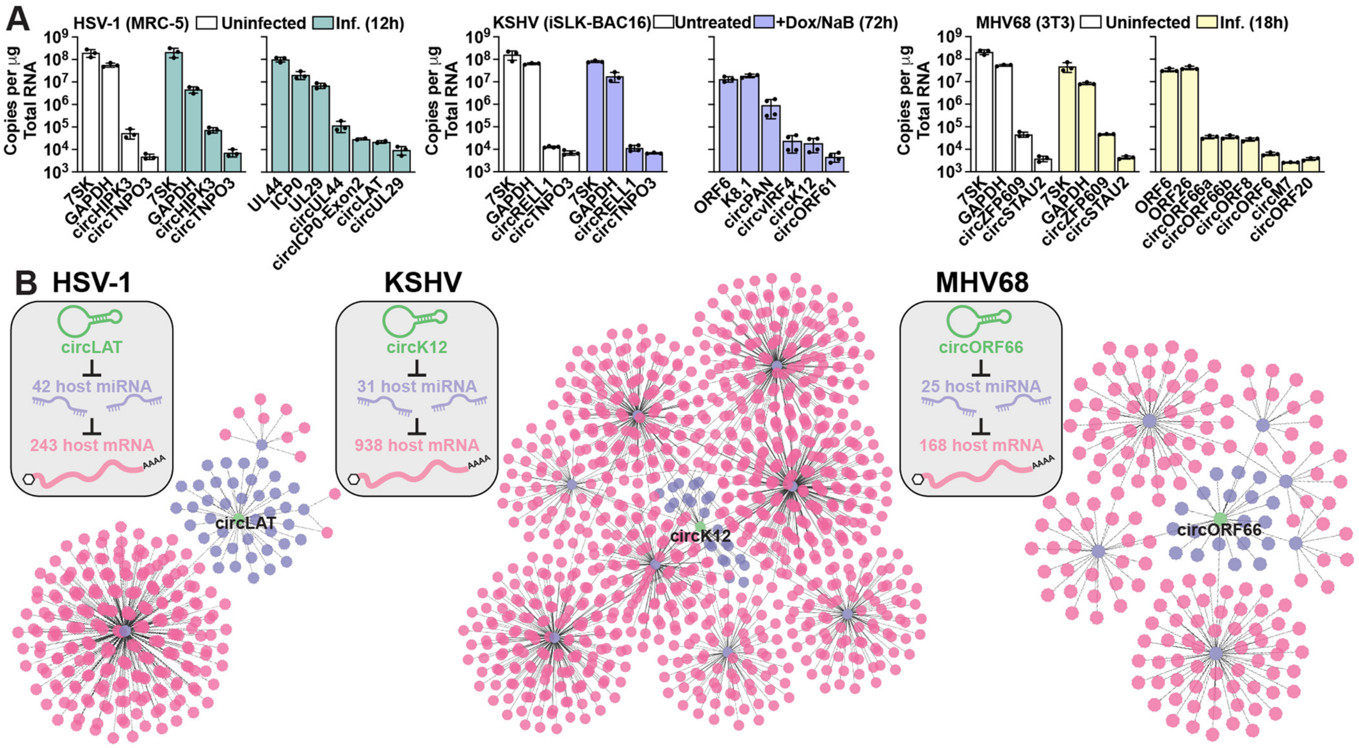

C **IPA Core Analysis** *(based on predicted mRNA targets)*

CircRNA	Pathway	p-value
circLAT	**Aryl Hydrocarbon Receptor Signaling** (*AHR,AHRR,ALDH1A2,ALDH9A1,CDK2,CDK4,CDK6,CYP1B1,NFIC,RARG,RELA,SP1,TRIP11*)	1.12E-8
	**Senescence Pathway** (*AKT2,ARAF,CDK2,CDK4,CDK6,CHP1,DHCR24,E2F5,ELF4,ELK3,MAPK14,NFATC1,PHF19,RASSF5,SMAD5,SQSTM1*)	2.24E-7
	**ID1 Signaling Pathway** (*AKT2,ARAF,CAV1,CCN2,CDK4,DVL2,EGR1,GSK3B,MAPK14,PGF,RHOC,SMAD5,STAT3*)	4.27E-7
circK12	**CLEAR Signaling Pathway** (*AKT3,ATF2,ATP6AP1,ATP6V0B,ATP6V0E2,ATP6V1E1,ATP6V1E2,ATP6V1F,ATP6V1G2,H2BW2,KRAS,MAP4K3,MAPK11,MAPK13,MAPK6, NTRK3,PIP4P1,PRKAB2,PRKAG1,PRKCD,YWHAE*)	7.94E-3
	**mTOR Signaling** (*AKT3,EIF3I,EIF4G3,FKBP1A,GPLD1,KRAS,PLD6,PRKAB2,PRKAG1,PRKCD,RHOB,RHOJ,RND3,RPS15A,RPS21*)	2.63E-2
	**Role of JAK family kinases in IL-6-type Cytokine Signaling** (*ADAM17,BCL2L1,CLCF1,MAPK11,MAPK13,OSMR,PIAS3*)	4.27E-2
circORF66	**Integrin and Thrombin Signaling** (*Cdc42,Ppp1r12b*)	4.37E-2

Figure EV3. Herpesvirus circRNA copy levels and in silico interaction networks.

(**A**) Copy level quantitation of viral circRNAs ($n = 3$–4). RNA was collected from the models described in Fig. 1. cDNA was quantified using digital droplet PCR (ddPCR) and convergent (linear transcripts) or divergent (circular transcripts) primers. Data is normalized to the total amount of RNA in the reverse transcription reaction and plotted as copies per µg total RNA. (**B**) In silico circRNA–miRNA-mRNA interaction networks for circLAT (HSV-1), circK12 (KSHV), and circORF66 (MHV68) variants highlighted in Fig. 1C, D. (**C**) Putative downstream mRNA targets were used to perform overrepresentation analysis. Pathway hits, mRNA targets present, and p values are given. Data Information: In column bar graphs, data points are biological replicates, bar maxima are the average, error bars are standard deviation. Ingenuity Pathway Analysis (IPA) core analysis *P* values are calculated using a Fisher's Exact Test.

 © The Author(s)

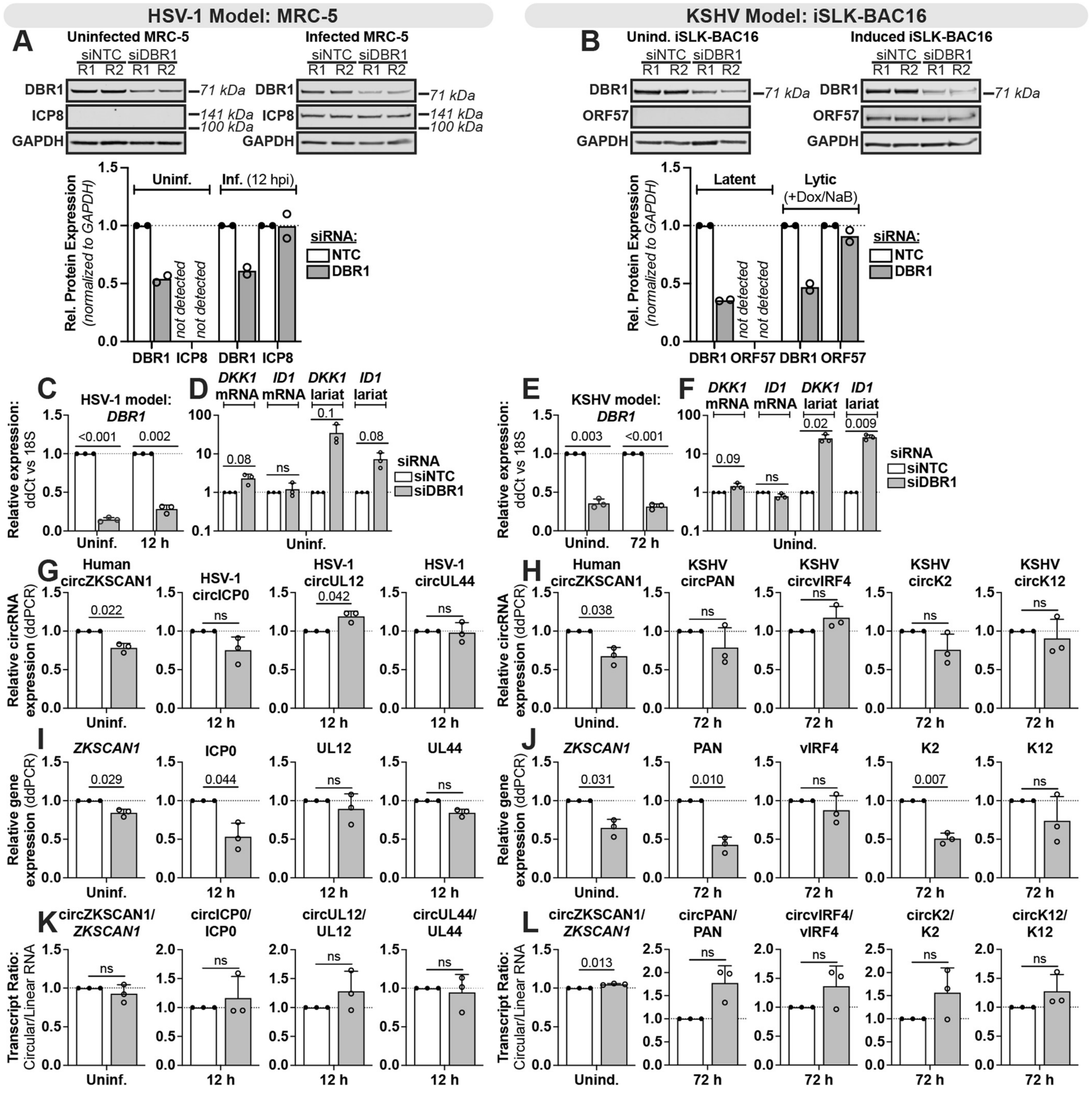

Figure EV4. Viral circRNAs are refractory to RNA lariat debranching enzyme depletion.

(**A**, **C**, **D**, **G**, **I**, **K**) MRC-5 were transfected with siRNAs targeting RNA lariat debranching enzyme (*DBR1*) or a nontargeting control (NTC) for 2 days. MRC-5 were mock (Uninf.) or HSV-1 infected for an additional 12 h. (**B**, **E**, **F**, **H**, **J**, **L**) iSLK-BAC16 were transfected with siRNAs targeting *DBR1* or a NTC for 24 h. Subsequently, cells were treated with vehicle (Unind.) or Dox and NaB for 72 h (72 h). (**A**, **B**) Protein expression was assessed by immunoblotting and quantified relative to a loading control (GAPDH). (**C**, **F**) Host transcripts were quantified by qPCR relative to the reference gene (18S). (**G–L**). ddPCR quantitation using divergent (circRNA) or convergent (gene) primers. Data Information: In column bar graphs, data points are biological replicates ($n = 2$–3), bar maxima are the average, for $n > 2$ error bars are standard deviation. All data is relative a paired siNTC sample. If $n \geq 3$, two-tailed paired t tests were performed, P values < 0.05 are labeled and not significant (ns) indicates P values ≥ 0.05. Source data are available online for this figure.

© The Author(s)

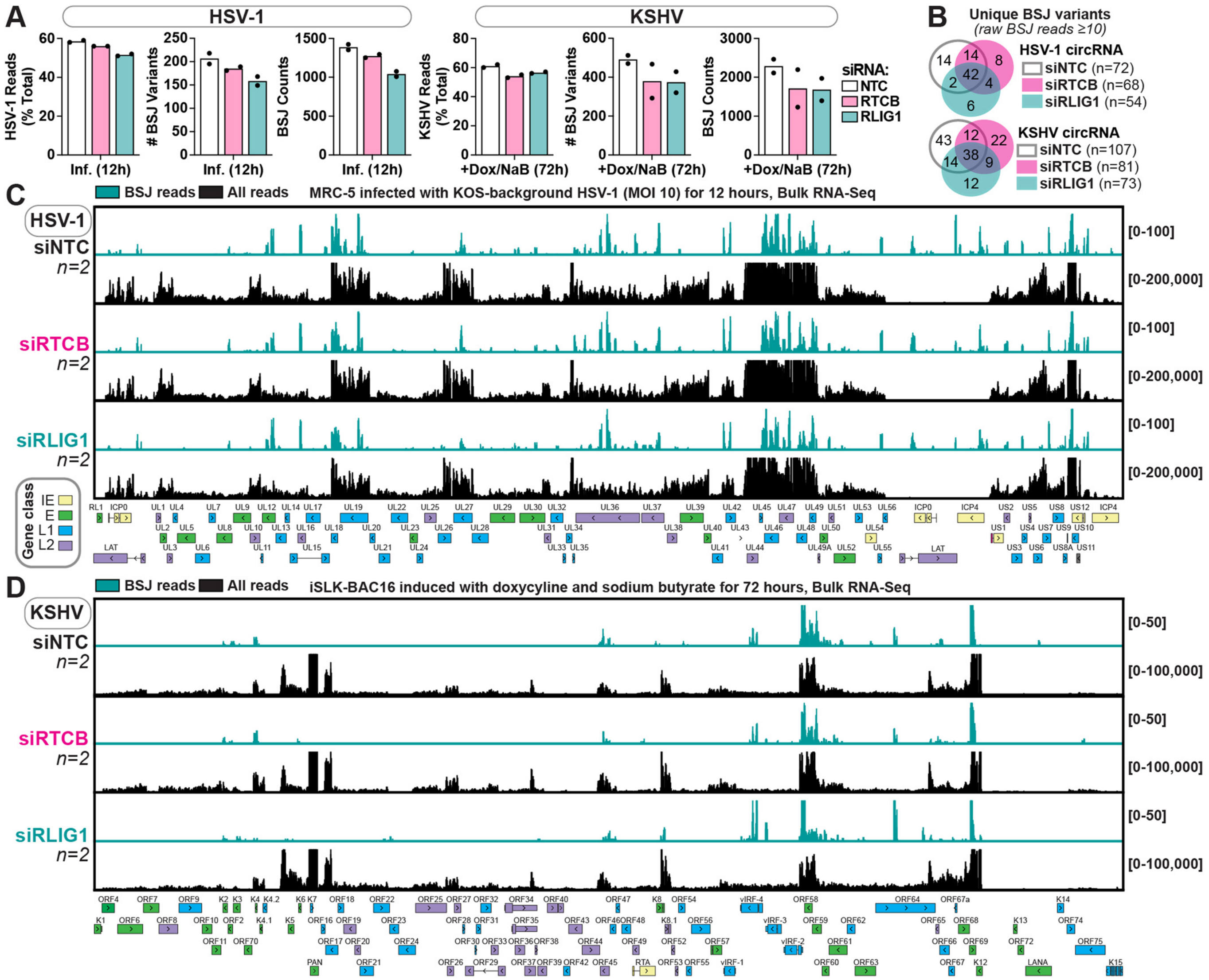

Figure EV5. Quantitation of viral RNAs after RNA ligase depletion.

(**A–D**) RNA-Seq data from infection models in Fig. 5. (**A**) RNA-Seq overview for all viral mapped reads (MR), as percent total reads (% Total). The number of unique BSJ variants and total BSJ counts is reported for high-confidence viral circRNAs. (**B**) Overlapping identity of high-confidence viral circRNAs, includes only BSJ with ≥10 reads per sample. (**C**, **D**) Visualization of high-confidence HSV-1 and KSHV circRNAs. Green and black traces include circular (back-spliced reads) and linear (non-chimeric) reads, respectively. Traces are the sum of raw BSJ or linear read values for biological duplicates. *Y* axis minimum and maximum values are shown on the right. Viral genes are shown below and labeled by gene class as IE (yellow), E (green), L1 (blue), and L2 (purple). Data Information: RNA-Seq ($n = 2$) was performed and high-confidence circRNAs were called using CHARLIE. In column bar graphs, data points are biological replicates, and bar maxima are the average.

 © The Author(s)
